# Concurrent Alterations in DNA Methylation and RNA m6A Methylation During Epigenetic and Transcriptomic Reprogramming Induced by Tail Docking Stress in Fat-Tailed Sheep

**DOI:** 10.3390/ani16030481

**Published:** 2026-02-04

**Authors:** Jian Zhang, Yannan Ma, Shuzhen Song

**Affiliations:** 1 Animal Husbandry, Pasture and Green Agriculture Institute, Gansu Academy of Agricultural Science, Lanzhou 730070, China; zhang2024@gsagr.cn; 2 College of Life Sciences, Northwest Normal University, Lanzhou 730070, China; mayannan@nwnu.edu.cn

**Keywords:** tail docking, fat-tailed sheep, DNA methylation, RNA m6A methylation, epigenetic regulation

## Abstract

Tail docking, serving as an important management intervention in animal husbandry, plays a significant role in regulating tail fat deposition and improving production performance and health status in fat-tailed sheep. This study systematically revealed the reprogramming effects of tail docking on the epigenetic landscape and transcriptome of fat-tailed sheep by integrating whole-genome bisulfite sequencing (WGBS) and RNA m6A methylated immunoprecipitation sequencing (MeRIP-seq). At the DNA level, the tail-docked group exhibited a pronounced trend of hypomethylation across multiple functional genomic regions, including promoters, exons, and introns. Differentially methylated regions (DMRs) were significantly enriched in pathways related to tissue development and stress response, such as the Hippo signaling pathway and adherens junctions. Pyrosequencing validation of the promoter region of the key gene DGAT1 further confirmed the reliability of the WGBS data. At the RNA level, RNA m6A modifications showed an overall up-regulated pattern: the tail-docked group displayed higher numbers of m6A peaks, greater total peak length, and increased genomic coverage compared to the control group, along with better overall prediction of modification sites. Genes associated with differential m6A peaks were closely related to processes such as stem cell pluripotency and cytoskeleton regulation. qPCR validation of several methylation-related enzyme genes (e.g., METTL3, FTO, YTHDF1) yielded results consistent with the sequencing trends. Through integrated analysis of DNA methylation and RNA methylation, we identified 143 genes with concurrent changes in methylation and mRNA expression, among which 41 genes were regulated by both DNA and RNA methylation. These genes were primarily enriched in the adherens junction pathway. Notably, two core genes CITED4 and ZNF644 showed significant changes across all three levels: DNA methylation, RNA methylation, and mRNA expression. This study systematically elucidates the epigenetic mechanism by which tail docking stress induces coordinated DNA hypo-methylation and RNA m6A hyper-methylation to regulate transcriptomic reprogramming in response to environmental intervention. The findings provide novel insights into the molecular basis of trait formation in livestock.

## Simple Summary

This study examined the molecular mechanisms underlying the improved health and productivity observed in fat-tailed sheep following tail docking—an economically important breed in which tail fat functions as an adaptive energy reserve. By analyzing post-procedure molecular changes, we found that tail docking decreases DNA methylation while elevating RNA m6A methylation. These coordinated epigenetic adjustments reprogram genes associated with tissue development and stress response, thereby facilitating physiological adaptation. Our findings elucidate the molecular basis of this adaptive process and offer valuable insights for optimizing breeding and management approaches to enhance both animal welfare and production efficiency.

## 1. Introduction

Fat-tailed sheep constitute a significant component of global ovine genetic resources, accounting for approximately 25% of the world’s sheep population [1]. The distinctive fat-tail phenotype is the result of long-term natural selection and artificial domestication, representing a stable and heritable trait. Historically, the caudal fat depot served as a critical metabolic energy reservoir during periods of food scarcity in winter and spring, particularly under harsh seasonal fluctuations and migration challenges [2]. Additionally, the enlarged tail surface area may confer benefits in wool production. However, with the shift towards intensive housing systems, the original adaptive functions of tail fat for energy maintenance and increasing body surface area have become obsolete [3]. Instead, excessive caudal fat deposition now reduces feed efficiency and impairs carcass quality. Consequently, minimizing tail fat deposition and improving carcass traits are paramount for enhancing the economic viability of fat-tailed sheep farming.

The Lanzhou Fat-tailed sheep is a characteristic long-fat-tailed breed. Its adipose tail naturally hangs down to the hock, presenting a distinct fan-shaped appearance. In castrated adult individuals, tail fat accounts for approximately 13.23% of the carcass weight. This long and bulky tail increases the risk of fecal contamination and myiasis [4,5], and poses practical challenges during breeding, lambing, slaughter, and shearing operations [6]. Our previous studies have demonstrated that tail docking in Lanzhou Fat-tailed sheep significantly reduces tail fat weight, overall fat content, and adipocyte diameter compared to undocked counterparts. It also alters the proportional area of lipid droplets within the tail adipose tissue, indicating that docking effectively remodels the body’s fat deposition pattern [7]. Furthermore, tail docking influences the mRNA expression levels of key genes involved in lipogenesis and lipolysis: in the tail fat of docked Lanzhou Fat-tailed sheep, the mRNA expression of SCD, LEP, and PLIN1 was significantly upregulated, while FAS expression was markedly downregulated.

Recent studies have identified several key genes involved in lipid metabolism in fat-tailed sheep that are regulated by DNA methylation. The methylation status of these genes influences their expression levels, thereby regulating adipocyte growth, differentiation, and lipid deposition. Research in model organisms has provided direct evidence for the central role of key DNA methylation enzymes in lipid metabolic regulation. For instance, knockout of DNMT3B in adipocyte precursor cells has been shown to significantly increase energy expenditure and effectively ameliorate diet-induced obesity and insulin sensitivity in female mice, revealing a clear sexual dimorphism in this regulatory effect [8]. In human population studies, the study pinpointed specific epigenetic modifications in peripheral blood, notably previously unrecognized CpG sites, which exhibited robust associations with circulating levels of both high-density and low-density lipoprotein cholesterol. These studies have also elucidated how behavioral factors like smoking and alcohol consumption modify these epigenetic marks, providing a population-level research framework for identifying epigenetic markers related to lipid metabolism [9]. Furthermore, “epigenetic scores” for metabolic traits such as body mass index (BMI) and body fat percentage, constructed using DNA methylation data, have demonstrated good predictive performance across multiple human cohorts. This offers a feasible methodological reference for predicting fat-related phenotypes via epigenetic markers [10]. Beyond DNA methylation, N6-methyladenosine (m6A) modification, an important epitranscriptomic regulatory mechanism, plays a gradually elucidated role in regulating lipid metabolism and environmental stress responses. Notably, in sheep models, recent research has confirmed that m6A modifications mediated by METTL3 and FTO can regulate the expression of key lipid metabolism genes such as FABP4 and ATGL, thereby influencing lipid deposition and modulating the response to heat stress in Hu sheep [11]. This study currently stands as the sole direct empirical investigation exploring the relationship between m6A and lipid metabolism using a sheep model.

Collectively, studies in model organisms, human populations, and target livestock species have confirmed the central role of DNA methylation and m6A modification in the regulation of lipid metabolism, revealing both conserved and species-specific regulatory mechanisms across different species. While existing evidence indicates that tail docking in Lanzhou Fat-tailed sheep alters body fat distribution patterns and the expression of related lipid metabolism genes, the specific mechanism underlying this docking-induced fat redistribution remains unclear. Therefore, this study aims to elucidate the epigenetic and transcriptomic reprogramming mechanisms by which tail docking regulates fat deposition in Lanzhou Fat-tailed sheep through DNA and m6A methylation. This will be achieved by investigating changes in methylation levels, expression of methylation-related genes, and specific gene methylation sites in adipose tissue before and after docking. The findings are expected to provide a scientific basis for epigenetic breeding strategies aimed at cultivating high-quality meat sheep.

## 2. Materials and Methods

### 2.1. Animals, Feeding Management, and Sample Collection

This study was conducted at the breeding base of Ruilin Technology Co., Ltd. in Yongjing County, Gansu Province, China (35°96′ N, 103°29′ E; altitude 1900 m). Characterized by a temperate climate, the region experiences an average annual temperature of 9.0 °C and receives approximately 300 mm of precipitation per year. Six 5-day-old male Lanzhou Large-tailed lambs with clear pedigrees and comparable body weights (4.1 ± 0.12 kg) were selected and randomly assigned to a control group (Group C) and a tail-docking treatment group (Group T), with three lambs per group. At 5 days of age, lambs in the treatment group underwent rubber-ring tail docking between the 3rd and 4th caudal vertebrae; Prior to the procedure, 2% lidocaine hydrochloride was applied at the docking site to minimize acute pain. Following docking, lambs were monitored daily for signs of distress, infection, or complications until the rings detached naturally after approximately 10 days (i.e., at 15–20 days of age) and full wound healing was observed (2% Lidocaine Hydrochloride). Tissues obtained during this initial docking procedure were not used in subsequent experiments, while lambs in the control group received no treatment. All lambs were reared under standardized environmental conditions (uniform temperature, lighting, and ventilation) and were provided with mixed concentrate feed throughout the trial. After a 300-day experimental period (reaching 305 days of age), all animals were fasted for 12 h and deprived of water for 3 h, then euthanized by intravenous injection of pentobarbital sodium (25 mg/kg) and slaughtered. Tail adipose tissue was collected, aliquoted into cryovials within 1 min, and preserved in liquid nitrogen before long-term storage at −80 °C. Upon returning to the laboratory, the same batch of tail adipose tissue samples collected at 305 days of age was immediately used for WGBS, RNA methylation sequencing, pyrosequencing validation, and quantitative real-time PCR (qPCR) analysis. The study was conducted in strict accordance with the Guidelines for the Care and Use of Experimental Animals in China and was approved by the Animal Ethics and Welfare Committee of the Institute of Animal Science, Grassland and Green Agriculture, Gansu Academy of Agricultural Sciences.

### 2.2. DNA Methylation Sequencing

#### 2.2.1. Bisulfite Treatment and Library Construction

Genomic DNA was randomly fragmented into small segments via ultrasonic shearing. Target DNA fragments (approximately 0.7–1.2 times the desired size) were then enriched through a stringent two-step size selection using LC Beads (Beckman Coulter, Brea, CA, USA). This purification procedure involved binding DNA to the beads, discarding the supernatant containing off-target fragments, followed by repeated washing with 80% ethanol to remove impurities. After drying, high-purity DNA fragments were eluted using nuclease-free water. Subsequently, the purified DNA fragments were subjected to bisulfite conversion using the EZ DNA Methylation-Gold™ Kit (Zymo Research, Irvine, CA, USA). Briefly, DNA was mixed with CT conversion reagent and denatured at 98 °C, followed by an extended incubation at 64 °C in a PCR thermal cycler. The conversion products were purified using a spin column: the sample was mixed with binding buffer, loaded onto the column, and bound to the membrane by high-speed centrifugation. The column was then sequentially washed, desulfonated, and washed again. Finally, the converted single-stranded DNA was eluted with elution buffer. The eluate was stored at low temperature, and an aliquot was taken for single-stranded DNA quantification to determine the optimal number of PCR amplification cycles for subsequent steps.

#### 2.2.2. Library Amplification and Final Purification

The conversion product was heat-denatured and immediately placed on ice to prevent reannealing. Subsequently, an enzymatic mixture was used to perform a “3′-end repair and adapter ligation” reaction, which repairs the 3′ ends of single-stranded DNA while simultaneously ligating the first sequencing adapter. Next, a second-strand synthesis reaction mix was added to synthesize the complementary strand using the first strand as a template, regenerating double-stranded DNA, which was immediately purified using LC Beads at a 1.2× ratio. Following this, a “5′-end adapter ligation” step was carried out to attach the second sequencing adapter to the opposite end of the DNA fragment, thereby constructing a complete library scaffold with adapters on both ends. The product from this step was similarly purified using LC Beads at a 1.0× ratio. Unique barcode-indexed amplification primers were then directly added to the ligation product, and a PCR amplification system was prepared. The amplification program began with an initial denaturation at 98 °C, followed by a defined number of exponential amplification cycles determined based on the initial DNA input. The final PCR product was stringently purified using LC Beads at a 0.8× ratio to remove impurities such as primer dimers, enzymes, and salts. The purified library was eluted in Tris buffer and transferred to a fresh tube. Finally, the library was accurately quantified using a Qubit fluorometer (Thermo Fisher Scientific, Waltham, MA, USA) to confirm that its concentration met the requirements for sequencing. Qualified libraries were stored at −20 °C until ready for high-throughput sequencing.

#### 2.2.3. High-Throughput Sequencing and Alignment Analysis

Using a paired-end 150 bp strategy, qualified libraries were subjected to sequencing on the Illumina NovaSeq 6000 platform (Illumina, San Diego, CA, USA). The target sequencing depth for each sample was set at 30-fold genome coverage. Quality control and adapter trimming on the raw sequencing data were conducted with Fastp (v0.23.0) to discard substandard reads. The cleaned reads were then aligned to the sheep reference genome (Oar_v4.0) using Bismark (v0.23.0), a software specifically designed for mapping bisulfite-treated sequences. During alignment, a maximum of two mismatches was permitted. PCR duplicates were removed using the deduplication tool integrated within Bismark. The bisulfite conversion efficiency was assessed either by aligning reads to the spiked-in unmethylated lambda phage DNA sequence or by calculating the C-to-T conversion rate at non-CpG cytosines (CHH and CHG contexts). The conversion efficiency for all samples exceeded 99.5%.

#### 2.2.4. DNA Methylation Analysis and Differentially Methylated Region (DMR) Identification

The methylation level at each CpG site, defined as the ratio of reads supporting methylation to the total coverage reads, was calculated using the Bismark methylation extractor. To identify DMRs, the genome was first partitioned into consecutive 200 bp sliding windows with a 50 bp step size. Only windows with a coverage depth of ≥5× in at least three samples per group were retained for downstream analysis. Differential methylation between the tail-docked and control groups was assessed using the R package DSS (v2.44.0). A window was considered differentially methylated if it met the following criteria: an absolute difference in the average methylation level (Δβ) ≥ 0.15, and a false discovery rate (FDR) < 0.05 after Benjamini–Hochberg correction for multiple testing. Significantly differentially methylated windows that were adjacent (spacing < 500 bp) were subsequently merged into a single DMR.

### 2.3. RNA Methylation Sequencing

#### 2.3.1. RNA Fragmentation and Immunoprecipitation

Following total RNA extraction from tissues, RNA integrity was assessed by agarose gel electrophoresis. The RNA was then precisely quantified using a Qubit 2.0 Fluorometer (Thermo Fisher Scientific, Waltham, MA, USA) (total amount ≥ 10 μg), and its quality was verified on an Agilent 2100 Bioanalyzer (Agilent Technologies, Beijing, China) to ensure a RNA integrity number (RIN) ≥ 7.5, thereby guaranteeing the reliability of subsequent library construction. The qualified RNA was subjected to fragmentation using RNA Fragmentation Reagents. Briefly, fragmentation was performed at 70 °C for 10 min to generate RNA fragments ranging from approximately 100 to 300 nucleotides, followed by purification via ethanol precipitation. A portion of the fragmented RNA was saved as the “Input control” for subsequent sequencing comparison. For immunoprecipitation, Protein A/G magnetic beads were washed with IP buffer (containing 150 mM NaCl, 10 mM Tris-HCl, pH 7.5) and then incubated with an m6A-specific antibody (5 μg, Millipore, cat# ABE572; Merck KGaA, Darmstadt, Germany) for 2 h at 4 °C to allow antibody conjugation. Subsequently, the fragmented RNA was added to the antibody-bead complex and incubated with rotation for 4 h at 4 °C to facilitate specific binding of m6A-modified RNA fragments. Following the binding reaction, the beads were subjected to three washes with ice-cold IP buffer to strip away any unbound RNA. The m6A-modified RNA was then eluted from the beads by incubation with free N6-methyladenosine for 1 h at 4 °C. The eluate was collected, and the RNA was purified by phenol-chloroform extraction, yielding enriched m6A-containing RNA fragments.

#### 2.3.2. Library Construction and Sequencing Analysis

Libraries were constructed from both the Input control and immunoprecipitated (IP) RNA samples using the SMARTer Stranded Total RNA-seq Kit. Following reverse transcription and cDNA synthesis, AMPure XP beads were employed for size selection to obtain fragments within the optimal size range. Library quality was rigorously assessed through a two-step procedure: initial quantification using Qubit 2.0, followed by analysis on an Agilent 2100 Bioanalyzer to verify the size distribution. Finally, the effective library concentration was precisely determined by qPCR, with a required threshold of >2 nM. Qualified libraries from both groups were pooled in an equimolar ratio and subjected to paired-end 150 bp (PE150) sequencing on an Illumina NovaSeq platform (Illumina, San Diego, CA, USA). Raw sequencing reads were first processed for quality control. Adapter sequences and low-quality bases were trimmed using trimmomatic (v0.39). The cleaned reads were then aligned to the reference genome using the HISAT2 aligner (v2.2.1), followed by filtering to remove multi-mapping and low-quality alignments. For the identification of m6A modification sites (peak calling), the exomePeak R package (v2.0) was utilized. Enriched m6A peaks across the genome were identified by comparing the read coverage between the IP and Input control samples. Significant peaks were defined using thresholds of |log_2_(fold change)| ≥ 1.5 and an adjusted *p*-value (FDR) < 0.05. Sequencing data from the Input control sample served as standard RNA-seq data for transcriptome profiling. Gene expression levels were quantified by generating raw read counts using featureCounts. Differential expression analysis was performed using the DESeq2 package (v1.38.0) in R. The median-of-ratios method within DESeq2 was applied for normalization. No significant batch effects were identified or corrected for in this analysis. A dual-threshold criterion (|log_2_ fold change| ≥ 1 and FDR-adjusted *p* < 0.05) was applied to determine statistically significant differentially expressed genes (DEGs). The called m6A peaks were subsequently annotated, and analyses of their genomic distribution, consensus motif, and differential methylated regions (DMRs) were performed, enabling integrated analysis of m6A methylation and gene expression profiles.

### 2.4. Genomic DNA Methylation Analysis by Pyrosequencing

Genomic DNA was extracted using the QIAGEN DNA extraction kit (QIAGEN, Hilden, Germany). DNA quality was verified by spectrophotometry, with concentrations ≥ 50 ng/μL and OD260/280 ratios between 1.8 and 2.0, and integrity was confirmed by agarose gel electrophoresis showing clear bands without degradation. Bisulfite conversion was performed using the EpiTect Bisulfite Kit, which converts unmethylated cytosine to uracil under the following thermal conditions: 95 °C for 5 min, 60 °C for 25 min, 95 °C for 5 min, 60 °C for 85 min, followed by hold at 20 °C. The converted DNA was purified and served as the template for subsequent PCR amplification. Bisulfite conversion efficiency was verified to be >99% for each sample by analyzing the conversion rate at non-CpG cytosine positions within the assayed amplicons. PCR was carried out in a 50 μL reaction mixture containing GC buffer, 1 μL each of forward and reverse primers (50 μM), 2 μL of bisulfite-converted template, and 0.2 U of Taq polymerase. Primers for the target region in the DGAT1 promoter amplified a fragment containing CpG dinucleotides for analysis. The amplification program consisted of an initial denaturation at 95 °C for 3 min; 35–45 cycles of 94 °C for 30 s, 56 °C for 30 s, and 72 °C for 1 min; and a final extension at 72 °C for 7 min. If non-specific bands were observed, the target fragment was purified using the QIAquick Gel Extraction Kit to ensure amplification specificity. For pyrosequencing, 10 μL of biotin-labeled PCR product was bound to streptavidin-coated magnetic beads, and single-stranded DNA templates were obtained following alkaline denaturation. Pyrosequencing was performed on a PyroMark Q48 instrument using sequential dispensing of dNTPs (with dATPαS replacing dATP). The enzymatic cascade involving DNA polymerase, ATP sulfurylase, luciferase, and apyrase generated light signals proportional to pyrophosphate (PPi) release upon nucleotide incorporation. Methylation levels were quantified based on peak heights in the resulting pyrogram. Each sample was analyzed with three technical replicates. Data were accepted only if the pyrosequencing quality control metrics passed the instrument’s standards, and the average coefficient of variation across technical replicates for the CpG sites was <10%.

### 2.5. Real-Time Quantitative PCR

TRIzol reagent (Thermo Fisher Scientific, Waltham, MA, USA) was utilized to extract total RNA, following the provided protocol. Briefly, 0.2 mL of chloroform was added per 1 mL of TRIzol, and the mixture was vigorously vortexed for 15 s, incubated at room temperature for 2–3 min, and then centrifuged at 12,000× *g* for 15 min at 4 °C. After being transferred and mixed with an equal volume of isopropanol, the aqueous phase was incubated at room temperature for 10 min to precipitate RNA. Subsequently, centrifugation (12,000× *g*, 10 min, 4 °C) was used to pellet the RNA. Finally, the pellet was washed with 75% ethanol and recovered by a final centrifugation (7000× *g*, 5 min, 4 °C). After air-drying for 5–10 min at room temperature, the RNA was dissolved in RNase-free water. RNA concentration and purity were determined spectrophotometrically. For cDNA synthesis, reverse transcription was performed using a standard 10 μL reaction system. RNA templates were first incubated at 70 °C for 3 min and immediately chilled on ice. After adding reverse transcriptase, the reaction was incubated at 37 °C for 60 min, followed by enzyme inactivation at 95 °C for 3 min. Quantitative real-time PCR (qPCR) was performed in a 20 μL reaction volume with β-actin (ACTB) as the endogenous control. The thermal cycling protocol comprised an initial denaturation at 93 °C for 2 min, followed by 40 cycles of denaturation at 93 °C for 1 min and annealing/extension at 55–60 °C for 1 min. Following amplification, a melting curve analysis was conducted to confirm amplification specificity and the absence of primer-dimers or non-specific products. All primers were designed to yield amplicons ranging from 80 to 300 bp in length, with theoretical Tm values of approximately 59–60 °C. Amplification efficiency (E = 10^−1/slope^ for each primer pair was determined using a standard curve generated from a serial dilution of pooled cDNA, with efficiencies between 90% and 110% considered acceptable. Each biological sample was analyzed in triplicate technical replicates.

Gene expression fold-changes were calculated according to the comparative 2^−ΔΔCt^ method. Prior to comparative analysis, data distribution normality and homogeneity of variances were verified using appropriate statistical tests. An independent samples *t*-test (SPSS 29, IBM, Armonk, NY, USA) was employed to compare expression levels across experimental groups, adopting a significance criterion of *p* < 0.05.

## 3. Results

### 3.1. Quality Control of DNA Methylome and m6A Epitranscriptome Sequencing

Raw sequencing data generated from WGBS underwent systematic quality assessment and control. The raw data output per sample ranged stably from 84.72 to 87.64 Gb, with base-call error rates all below the Q40 threshold (Supplementary Figure S1), indicating a base-calling accuracy of >99.99% and thus providing a high-confidence foundation for downstream preprocessing. The raw reads were further processed to obtain clean reads according to the following criteria: removal of reads containing adapter sequences; removal of reads in which more than 5% of bases were undetermined (N); and removal of low-quality reads (where >20% of bases had a Phred quality score ≤ 10). After preprocessing, the clean data volumes for the three control samples (C1, C2, C3) and the three treatment samples (T1, T2, T3) were as follows: C1 (72.72 Gb), C2 (76.20 Gb), C3 (75.89 Gb), T1 (76.55 Gb), T2 (76.66 Gb), and T3 (77.20 Gb). The percentage of valid reads retained ranged from 96.00% to 98.84%, indicating minimal data loss. Quality metrics showed that >98% of bases had an accuracy of ≥99% (Q20 > 98%), and >84.8% of bases achieved an accuracy of ≥99.9% (Q30 > 84.8%) (Table 1). The GC content across samples fell within 26.62% to 28.92%, consistent with the expected range for mammalian genomes. Summary statistics for WGBS quality control metrics: alignment rate, duplication rate, and bisulfite conversion rate (Supplementary Table S1). All samples demonstrated a consistently high bisulfite conversion rate of over 99.4%. The T group (T1–T3) exhibited a slightly higher total number of sequencing reads and average alignment rate (approximately 74.8%) compared to the C group. The duplication rate for all samples was below 10%, with the C group showing a marginally lower average duplication rate than the T group, indicating that data redundancy was well-controlled. As the sequencing depth increased (0→10→30×), the majority of CpG sites were covered at low depths (0–10×), while only a minimal proportion achieved coverage above 20× (Supplementary Figure S2). In the low-depth range (0–30), the percentage curves of the treatment groups (T1, T2, T3) exhibit markedly stronger fluctuations compared to the control groups (C1, C2, C3), with the T3 group showing the most pronounced variation (peak nearly 5%). This difference visually reflects the presence of M-bias. As sequencing depth increases (≥30), fluctuations in both sets of curves diminish and gradually converge. At depths ≥ 50, all curves decline steadily and approach zero, indicating that the effect of M-bias subsides with increasing depth (Supplementary Figure S3). Overall, the WGBS data exhibited excellent depth, accuracy, and completeness, fully meeting the criteria for high-quality methylome analysis and supporting reliable alignment and differential methylation analysis in subsequent steps.

In this study, MeRIP-seq analysis was performed on 12 samples (Table 2), yielding a total of 97.79 Gb of high-quality clean data. The raw sequencing reads per sample ranged from 56.40 to 66.20 million (RawReads), corresponding to 8.46–9.93 Gb of raw bases (RawBases). Following quality control, 49.32–57.37 million clean reads (CleanReads) were retained, representing 7.40–8.61 Gb of clean bases (CleanBases). The percentage of valid bases ranged from 82.76% to 94.15%, indicating efficient data filtering. Base quality assessment showed that the Q30 scores across all samples ranged from 84.99% to 93.26%. Notably, the Q30 values in the treatment groups (T1–T3; 92.48–93.26%) were consistently higher than those in the control groups (C1–C3) and their corresponding input controls (C1_input–C3_input, T1_input–T3_input; 84.99–88.97%), suggesting superior base-call accuracy in the treatment samples. Analysis of GC content revealed values between 56.85% and 63.87%. The input control samples (C1_input–C3_input, T1_input–T3_input) exhibited generally higher GC content (58.95–63.87%) compared with the corresponding immunoprecipitated samples from both control and treatment groups (56.85–59.05%), which is consistent with the expected GC distribution for this type of sequencing data and indicates no substantial sequence bias. Overall, the post-processing sequencing data demonstrate high reliability and are suitable for downstream analyses.

### 3.2. Distribution Analysis of DNA and RNA Methylation

**Figure 1:**
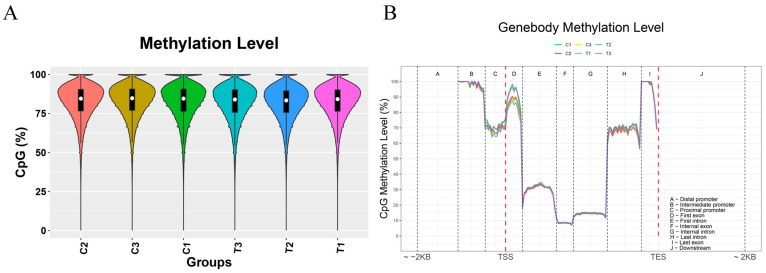
Summary of methylation distribution. (**A**) Analysis of methylation patterns across distinct genic compartments. (**B**) Assessment of global methylation levels.

The genome-wide average methylation levels were calculated for each sample (Figure 1A). All samples exhibited high uniformity and stability in CpG methylation, with control sample C1 and treated sample T3 showing relatively higher methylation levels, making them the two most distinct individuals in the dataset. To further investigate methylation patterns associated with genomic functional elements, we systematically analyzed the average methylation levels of cytosine sites in different sequence contexts across key genomic regions—including promoters, exons, introns, and downstream regions—within the interval spanning from 2 kb upstream of the transcription start site (TSS) to 2 kb downstream of the transcription termination site (TES) (Figure 1B). The results revealed highly consistent methylation distribution patterns across these functional regions among all samples, with no obvious inter-sample divergence, indicating a conserved regulatory landscape of DNA methylation in genomic functional domains across experimental groups.

RNA methylation peaks were identified using MeTDiff with a threshold of *p* ≤ 0.05, fold change (FC) ≥ 1.5 and FDR ≤ 0.05. Key parameters including peak number, length, and genomic coverage were quantified (Table 3). The control group contained 14,469 m6A peaks (genomic coverage: 2.22%), while the experimental group exhibited 15,325 peaks (coverage: 2.40%). Peak length distributions in both groups were highly skewed: median lengths were similar (~585–595 bp), but average lengths were substantially larger (~4000–4093 bp), indicating the presence of a limited number of exceptionally long peaks that inflate the mean.

Based on the variable prediction outcomes of methylation sites across samples, the identified sites were categorized into five confidence levels—Non, Low, Moderate, High, and Very High—and their proportional distribution was visualized using a stacked bar chart (Supplementary Table S2). Statistical analysis revealed that the experimental group contained a total of 1,237,848 methylation sites, of which 32,484, 101,739, 95,254, and 81,508 were classified as Very High, High, Moderate, and Low confidence, respectively, with 926,863 sites categorized as Non. In the control group, a total of 1,151,123 methylation sites were identified, including 30,706, 96,480, 89,741, and 76,617 sites in the Very High, High, Moderate, and Low confidence categories, respectively, and 857,579 Non sites. In summary, the total number of methylation sites in the experimental group increased by 86,725 compared with the control group. Furthermore, the counts of sites across all confidence levels, as well as the Non sites, were consistently higher in the experimental group. These findings suggest that the experimental condition may be associated with a greater abundance of methylation sites and an overall improved detection scale in site prediction.

**Figure 2:**
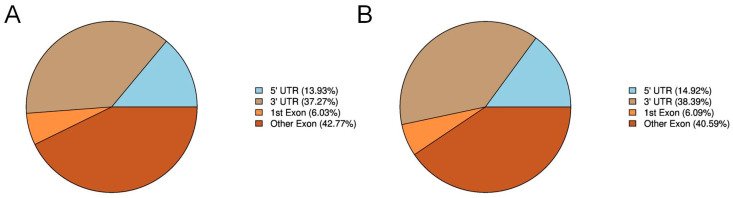
Analysis of RNA Methylation. (**A**) Distribution of methylation sites. (**B**) Annotation of peaks within genomic functional elements in the control group.

Annotation analysis of the detected RNA methylation peaks across gene functional elements revealed their distribution patterns. In the control group, m6A peaks were distributed as follows: 13.93% in the 5′ UTR, 37.27% in the 3′ UTR, 6.03% in the first exon, and 42.77% in other exons (Figure 2A). In the experimental group, the distribution was 14.92% in the 5′ UTR, 38.39% in the 3′ UTR, 6.09% in the first exon, and 40.59% in other exons (Figure 2B). Collectively, both the control and experimental groups exhibited an identical distribution pattern: other exons > 3′UTR > 5′ UTR > 1st exon. Furthermore, the proportional contributions of each functional element remained largely consistent between the two groups.

### 3.3. DMR Analysis

**Figure 3:**
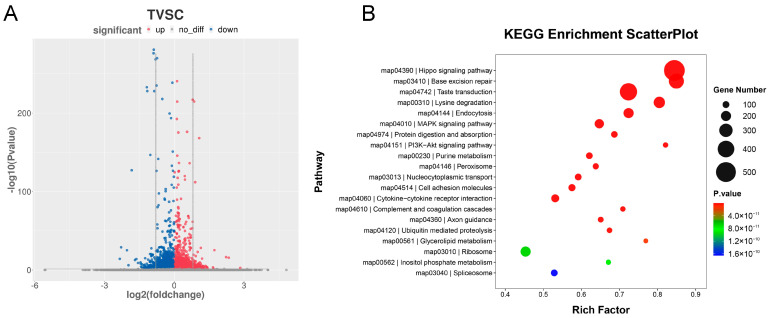
Analysis of DMRs. (**A**) Quantification of DMRs. (**B**) Volcano plot displaying DMRs.

To investigate differential methylation patterns between tail-docked and intact fat-tailed sheep, differentially methylated sites across distinct genomic regions—including promoters, exons, introns, intergenic regions, and CpG islands (CGIs)—were statistically analyzed. The results showed that the number of hypomethylated sites significantly exceeded that of hypermethylated sites in all examined regions. Specifically, promoter regions contained 3565 hypermethylated and 4858 hypomethylated sites; exons contained 6744 hypermethylated and 8346 hypomethylated sites; introns contained 26,745 hypermethylated and 35,593 hypomethylated sites; intergenic regions contained 126,960 hypermethylated and 184,568 hypomethylated sites; and CGIs contained 116 hypermethylated and 221 hypomethylated sites. These observations indicate that differentially methylated regions (DMRs) were predominantly hypomethylated in the tail-docked group relative to the intact group. ll DMRs were further analyzed using a threshold of FDR < 0.05 (Figure 3A). The analysis revealed that downregulated DMRs displayed higher statistical significance, although a large number of DMRs did not reach statistical significance. Functional enrichment analysis of genes associated with DMRs (Figure 3B) indicated that these genes were primarily enriched in pathways including the Hippo signaling pathway, base excision repair, taste transduction, lysine degradation, and endocytosis. In contrast, lower enrichment was observed for the ribosome pathway, inositol phosphate metabolism, and spliceosome pathway.

### 3.4. Pyrosequencing Validation of Methylation Changes at DGAT1 Loci

Based on the methylation profiles of the DGAT1 gene obtained from WGBS, seven primer pairs targeting its promoter region were designed (Table 4) to validate multiple CpG sites via pyrosequencing and conduct CpG-level analysis for the gene’s seven targeted regions (Assay 1S–7S), with validation results for the selected sequencing regions presented in Supplementary Figures S4–S10 and detailed CpG-level data summarized in Supplementary Table S3. The DNA methylation levels at CpG sites in the DGAT1 promoter region showed high consistency with the WGBS data. The pyrograms displayed stable baselines, well-resolved signal peaks (A, C, G, T) for each nucleotide incorporation, and a high signal-to-noise ratio, indicating efficient sequencing and reliable raw data suitable for downstream analysis.

### 3.5. Enrichment of Differentially m6A-Methylated Genes

Pathway enrichment analysis of the top 30 peak-associated genes was performed using the KEGG database. In the control group, enriched pathways in Cellular Processes primarily included endocytosis, autophagy–animal, apoptosis, and adherens junction. Environmental Information Processing was mainly enriched in the cGMP–PKG signaling pathway, TNF signaling pathway, Sphingolipid signaling pathway, Rap1 signaling pathway, and MAPK signaling pathway. Genetic Information Processing largely focused on Proteoglycans in cancer, Prostate cancer, Pathways in cancer, and Pancreatic cancer. Organismal Systems showed predominant enrichment in Parathyroid hormone synthesis, secretion and action, Neurotrophin signaling pathway, Longevity regulating pathway, and Axon guidance pathway (Supplementary Figure S11). In the experimental group, Cellular Processes were primarily concentrated in Focal adhesion, Endocytosis, Autophagy–animal, and Adherens junction. Environmental Information Processing was mainly enriched in the TNF signaling pathway, Sphingolipid signaling pathway, Ras signaling pathway, Rap1 signaling pathway, Phosphatidylinositol signaling system, and ErbB signaling pathway. Organismal Systems showed predominant enrichment in the Neurotrophin signaling pathway, Insulin signaling pathway, and Axon guidance pathway (Supplementary Figure S12).

### 3.6. Enrichment of Differential RNA m6A Methylation Peaks

**Figure 4:**
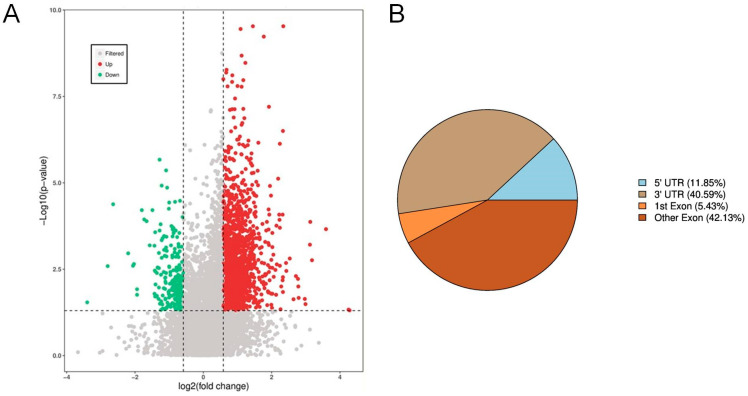
Enrichment Analysis of Differential RNA m6A Methylation Peaks. (**A**) Volcano plot of differential methylation peaks. (**B**) Annotation of differential peaks across genomic functional elements.

To systematically evaluate the genomic features of differentially methylated peaks, we quantified the number of peaks, total width, average width, and median width for each sample (Table 5). Using thresholds of *p* < 0.05 and |log_2_FC| > 0.58 for the T_Group-vs-C_Group comparison, a total of 2008 differential peaks were identified, comprising 1780 upregulated and 228 downregulated peaks. The total length of differential peaks was 6,961,677 bp, with an average length of 3466.97 bp and a median length of 448 bp. These metrics indicate an uneven genomic distribution of differential peaks, with the presence of several large peak regions. The differential peaks accounted for 0.27% of the genome, reflecting their relative specificity in genomic localization. The significance levels of upregulated peaks were generally higher than those of downregulated peaks, while a substantial number of methylation peaks did not meet the significance threshold (Figure 4A), highlighting the complexity of methylation regulation. Analysis of the distribution of differential peaks across gene elements revealed a strong enrichment in genic regions, predominantly in the 3′ UTR (40.59%) and other exons (42.13%), with notable proportions also observed in the 5′UTR (11.85%) and the first exon (5.43%) (Figure 4B). KEGG functional enrichment analysis of genes associated with differential peaks showed that within Cellular Processes, the most enriched pathways included Signaling pathways regulating pluripotency of stem cells, Regulation of actin cytoskeleton, and Endocytosis. Under Environmental Information Processing, significant enrichment was observed for the cGMP–PKG signaling pathway, TGF-beta signaling pathway, Sphingolipid signaling pathway, Rap1 signaling pathway, and NF-kappa B signaling pathway. In Organismal Systems, the top enriched terms were Leukocyte transendothelial migration, Fc gamma R-mediated phagocytosis, Chemokine signaling pathway, B cell receptor signaling pathway, and Axon guidance pathway (Supplementary Figure S13).

Given the inherent variation in gene expression levels and distributions across samples, genes were categorized into distinct intervals based on their FPKM values, and the number of genes expressed in each interval was calculated for each sample (Supplementary Table S4). In the FPKM 0–0.5 interval, the mean number of genes in the C_group (7861.00) was higher than that in the T_group (7476.67). In the FPKM 0.5–1 interval, the mean for the C_group (1156.00) was lower than that for the T_group (1237.67), indicating a higher average level in the T_group for this range. In the FPKM 1–10 interval, the mean for the C_group (6533.00) was lower than that for the T_group (6731.67), showing better average performance in the T_group. In the FPKM ≥ 10 interval, the mean for the C_group (4969.0) was also lower than that for the T_group (5073.0), with the T_group exhibiting a higher average value. In summary, within the FPKM 0–0.5 interval, the mean for the C_group was higher than that for the T_group, whereas in the FPKM 0.5–1, FPKM 1–10, and FPKM ≥ 10 intervals, the mean for the T_group was higher than that for the C_group. Collectively, compared with the C_group, the T_group contained fewer low-expression genes (FPKM 0–0.5) but more moderately and highly expressed genes (FPKM ≥ 0.5).

### 3.7. Pathway Enrichment Analysis of DEGs

**Figure 5:**
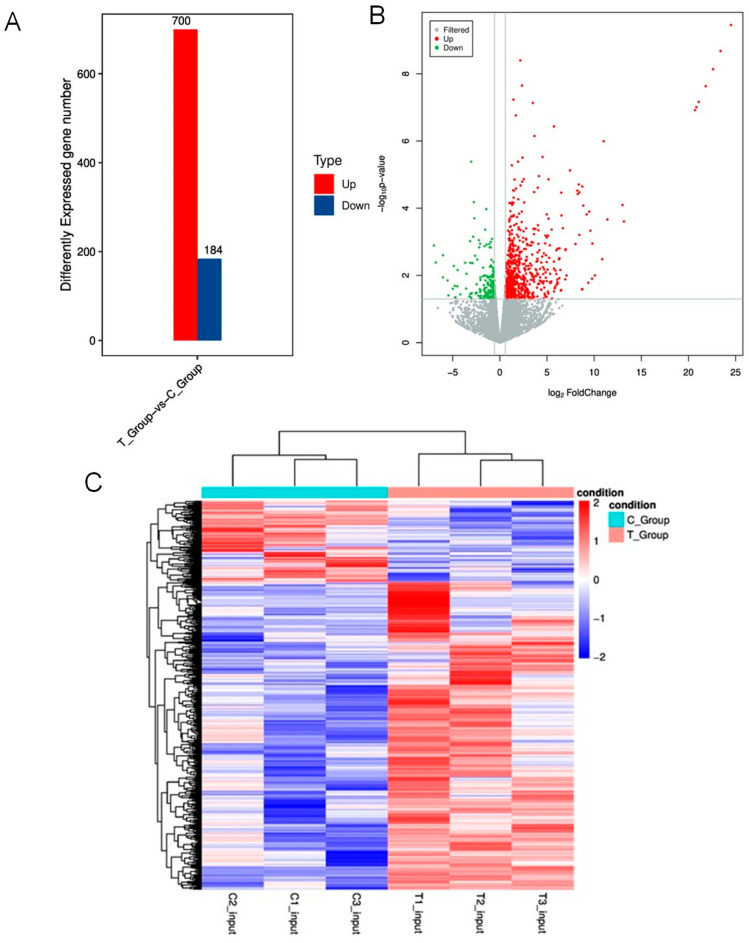
Functional Enrichment Analysis of Genes with Differential RNA m6A-associated Expression. (**A**) Quantification of DEGs. (**B**) Volcano plot of DEGs. (**C**) Hierarchical clustering analysis of DEGs across comparison groups.

Comparative analysis between the experimental and control groups, using thresholds of *p*-value < 0.05 and |log_2_FC| > 0.58, identified a total of 884 DEGs. This included 700 upregulated and 184 downregulated genes (Figure 5A). The magnitude of fold change was generally greater for upregulated DEGs than for downregulated ones, while a substantial number of genes did not reach the significance threshold (Figure 5B). This pattern indicates that tail docking induces a broad and asymmetric transcriptional response in fat-tailed sheep. Unsupervised hierarchical clustering of the DEGs showed clear separation between the experimental and control groups (Figure 5C). Consistent with the direction of differential expression, highly expressed genes were more abundant in the experimental group, whereas lowly expressed genes were less abundant compared to the control group. Pathway enrichment analysis of the DEGs revealed that within Cellular Processes, the most significantly enriched terms included Signaling pathways regulating pluripotency of stem cells, Phagosome, and Focal adhesion. Environmental Information Processing was primarily enriched in the Wnt signaling pathway, Hippo signaling pathway, ECM-receptor interaction, and Cell adhesion molecules (CAMs). In Organismal Systems, major enrichment was observed for Cardiac muscle contraction, Th1 and Th2 cell differentiation, Th17 cell differentiation, Protein digestion and absorption, and Antigen processing and presentation (Supplementary Figure S14). In summary, tail docking may trigger extensive transcriptomic reprogramming in fat-tailed sheep, potentially by modulating key biological processes such as cell fate determination, cell–cell interactions, and immune regulation.

### 3.8. Integrated Analysis of Differential Methylation and Gene Expression

**Figure 6:**
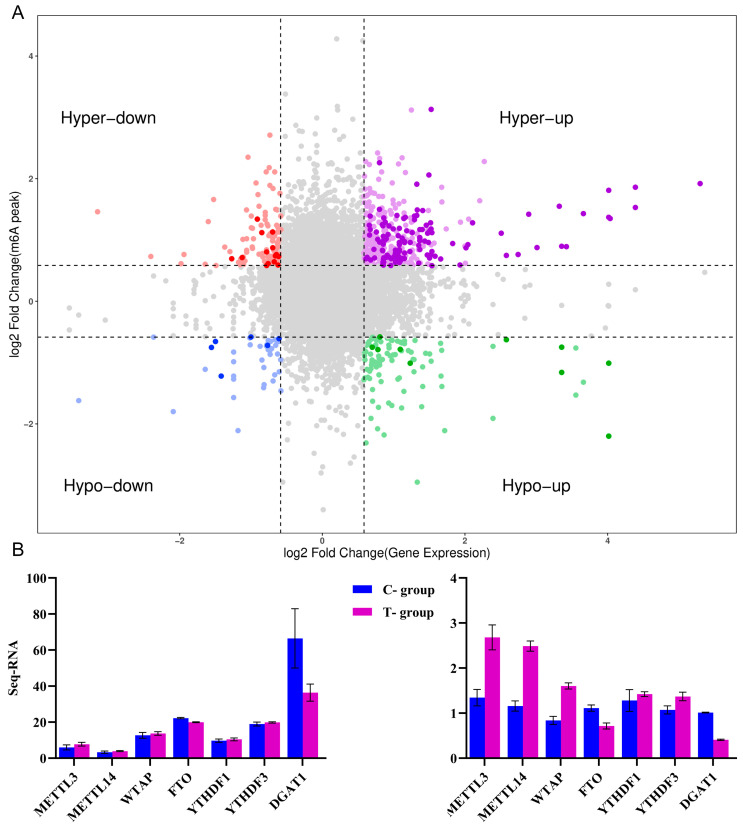
Integrated Analysis of Differential Methylation and Expression. (**A**) A four-quadrant scatter plot was generated to visualize the correlative relationship between differential methylation and differential gene expression. Gray dots represent genes not significant for both analyses; light-colored dots denote genes significant for only one parameter; dark-colored dots indicate genes significant for both differential methylation and expression. (**B**) Validation of mRNA expression levels by quantitative real-time PCR (qRT-PCR).

Integrated analysis of differential methylation and gene expression identified 151 genes exhibiting concurrent significant changes in both methylation levels and expression, revealing a complex regulatory relationship between their methylation status and transcriptional output (Figure 6A). Among these, 121 genes showed concomitant upregulation in both methylation and expression. Ranked by the magnitude of change, the top 10 genes in this category were: MYLK4, JPH1, NT5C1A, C25H10orf71, SCN4A, DUSP13, SRL, KLHL30, CALHM3, and SUSD4. Conversely, 14 genes displayed increased methylation alongside downregulated expression, ranked as follows: LOC101120850, OSMR, PIGU, MED7, FAM219A (36985795–36986044), FAM219A (36980778–36983905), OXSM, POLI, MRPS9, YTHDF2, KRCC1, ZNF839, GOLGA7, and ZNF503. Furthermore, 6 genes exhibited coordinated downregulation in both methylation and expression, ranked as: PPM1E, LOC101119976, SCN1A, PPARGC1B, SYN2, and DLAT. Ten genes showed decreased methylation but increased expression, ranked as: C25H10orf71 (42999823–43000170), C25H10orf71 (42992494–42997984), SRL (3422362–3422610), SRL (3420674–3421171), ANK1, ID2, CPE, SEMA3B, TWF2, and LOC101113020. To validate the sequencing data, seven key genes were selected for quantitative real-time PCR (qPCR) verification. These included three methyltransferase genes (METTL3, METTL14, and WTAP), one demethylase gene (FTO), and two m6A reader genes (YTHDF1 and YTHDF3) (primer sequences in Table 6). The qPCR results showed consistent expression trends with the sequencing data (Figure 6B), confirming the reliability and high credibility of the omics findings.

**Figure 7:**
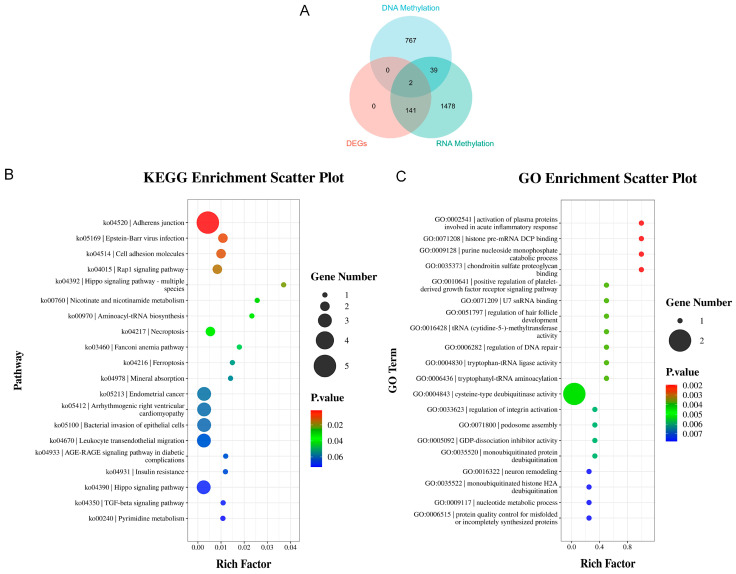
Integrated Analysis of DNA Methylation, RNA Methylation, and Differentially Expressed mRNAs. (**A**) Overlap analysis among methylation changes and differential expression. (**B**) GO biological process enrichment analysis of the overlapping genes. (**C**) KEGG pathway enrichment of the overlapping genes.

This study performed an integrated analysis of DNA methylation, RNA methylation, and differentially expressed mRNA (Figure 7A). The analysis identified 808 differentially DNA-methylated genes, 143 differentially expressed mRNAs (*p*-value < 0.05 and |log_2_FC| > 3.2), and 1660 differentially RNA-methylated genes. Among these, 41 genes exhibited concurrent changes in both DNA and RNA methylation. Furthermore, two genes, CITED4 and ZNF644, showed alterations across all three layers: DNA methylation, RNA methylation, and mRNA expression. Functional enrichment analysis was subsequently focused on genes exhibiting concurrent changes in both DNA and RNA methylation. Gene Ontology (GO) analysis indicated their primary involvement in biological processes and molecular functions such as the activation of plasma proteins involved in the acute inflammatory response, histone pre-mRNA DCP binding, purine nucleoside monophosphate catabolic process, and chondroitin sulfate proteoglycan binding (Figure 7B). KEGG pathway enrichment analysis of these overlapping genes revealed significant enrichment in the Adherens junction pathway, followed by the Epstein–Barr virus infection pathway, Cell adhesion molecules pathway, and the Rap1 signaling pathway (Figure 7C).

## 4. Discussion

Tail docking reduces tail fat deposition, improves feed conversion efficiency, and lowers the risk of diseases such as myiasis and tail rot. However, the epigenetic mechanisms triggered by tail docking remain poorly understood. In this study, we employed WGBS and methylated RNA immunoprecipitation sequencing (MeRIP-seq) to systematically investigate the impact of tail docking on the epigenetic landscape and transcriptome of fat-tailed sheep. Our findings reveal that tail docking induces a reprogramming characterized by the concurrent occurrence of hypomethylated DMRs predominating and RNA m6A hypermethylation, which were associated with transcriptomic changes in response to stress. This study provides novel insights into the epigenetic regulation of fat-tailed sheep and establishes a foundation for optimizing their production management.

High-quality sequencing data are essential for robust epigenetic [12] and transcriptomic analyses [13]. In this study, the WGBS data for each sample exhibited Q20 and Q30 scores exceeding 98% and 84.8%, respectively, indicating an extremely low base-calling error rate and high data accuracy. In the MeRIP-seq data, the Input samples showed slightly higher GC content compared with the immunoprecipitated (IP) samples, which may reflect preferential binding of the m6A antibody to specific RNA regions with moderate GC content. Input samples played a crucial role in reducing background noise and ensuring the specificity of m6A peak detection. Furthermore, peak-calling results demonstrated that the number, total length, and genomic coverage of m6A peaks were higher in the treated group than in the control group. However, it is important to consider that such metrics in MeRIP-seq can be influenced by technical variables including sequencing depth, IP efficiency, library complexity, and peak-calling thresholds. This interpretation is further supported by the classification of methylation sites based on confidence levels: the total number of methylation sites in the treated group exceeded that in the control group by 86,725, with consistently higher counts across all confidence categories.

CpG methylation is one of the most extensively studied epigenetic modifications, playing crucial roles in regulating gene expression, cell differentiation, and stress responses [14]. Analysis of DMRs in the tail-docked group revealed a pattern where hypomethylated regions significantly outnumbered hypermethylated regions across genomic features such as promoters, exons, and introns. This finding aligns with previous studies indicating that environmental stress often induces a shift toward DNA hypomethylation [15]. For instance, Panashe Kupakuwana et al. (2024) demonstrated that the liver exhibits molecular evidence of DNA hypomethylation under hypoxia/water stress [16]. Similarly, in Daphnia, the DNA methylation inhibitor 5-azacytidine leads to widespread hypomethylation [17]. These studies on DNA hypomethylation in response to physiological stressors suggest a mechanism that may facilitate the re-expression of genes required for adaptation, potentially stemming from the activation of DNA demethylases or the inhibition of DNA methyltransferase (DNMT) activity.

Genes associated with DMRs were functionally enriched in pathways such as the Hippo signaling pathway, base excision repair, and adherens junction. The Hippo pathway is a highly conserved signaling cascade that regulates cell proliferation, differentiation, organ growth, embryonic development, and tissue regeneration/wound repair [18]. Importantly, dysregulation of the Hippo pathway is closely linked to fat deposition and tissue development. For instance, in sheep models, YAP1 was found to promote the differentiation of adipose-derived stem cells into adipocytes by activating a negative feedback loop of the Hippo pathway (upregulating LATS2) [19]. Conversely, activation of YAP/TAZ in mature adipocytes can “dedifferentiate” them into precursor-like cells, leading to a significant reduction in fat mass and coordinating whole-body energy balance [20]. Phosphorylation-mediated inhibition of YAP/TAZ by LATS2 promotes adipocyte differentiation and inhibits proliferation, forming a fundamental mechanism by which this pathway regulates adipose development [21]. Furthermore, DNA methyltransferase DNMT3B and demethylase TET1 co-regulate the methylation status of the promoter region of LATS1, a key kinase in the Hippo pathway, thereby inactivating the pathway [22]. The enrichment of DMRs in the Hippo signaling pathway in our study suggests a hypothesis that tail docking may modulate tissue repair and re-establish tissue homeostasis by altering the methylation status of genes within this pathway. Studies have shown that the base excision repair capacity is specifically regulated during the differentiation of bone marrow mesenchymal stem cells into adipocytes, with upregulation of related proteins helping adipocytes resist DNA damage and maintain function [23]. The significant enrichment of the base excision repair pathway in our analysis could indicate that tail docking may induce DNA damage, prompting the organism to potentially activate relevant DNA repair pathways as a response.

The promoter region is a key regulatory element that drives gene expression, with transcription factor binding sites serving as core sequence determinants of its activity [24]. In this study, promoter regions contained 4858 hypomethylated and 3565 hypermethylated sites. DGAT1, a critical gene involved in triglyceride synthesis and fat deposition [25], exhibited hypomethylation in its promoter region, which could potentially lead to enhanced expression and thereby represent one possible mechanism influencing fat metabolism. This observation suggests that tail docking may activate the expression of a broader set of genes by reducing promoter methylation. This aligns with the findings of Panpan Guo et al., who reported that interfering with the DGAT1 gene in bovine preadipocytes significantly decreased triglyceride (TAG) content, thereby demonstrating the necessity of normal DGAT1 expression for bovine fat deposition [26]. Thus, the observed promoter hypomethylation of DGAT1 provides a plausible epigenetic explanation for the reduced fat deposition phenotype following tail docking. Pyrosequencing is a high-accuracy method for detecting DNA methylation at specific CpG sites and is widely used to validate WGBS results [27]. Employing pyrosequencing for validation was therefore crucial for ensuring the accuracy of DMR identification in this study. Similarly, Florence K. Crary-Dooley et al. used pyrosequencing to validate promoter methylation levels identified by WGBS, confirming the reliability of the WGBS data [28]. In future studies on fat regulation in fat-tailed sheep, the specific epigenetic mechanisms of DGAT1 warrant further investigation.

RNA m6A methylation is the most prevalent internal modification in eukaryotic mRNAs, playing key roles in regulating RNA splicing, stability, translation, and nuclear export [29]. In contrast to the observed pattern of hypomethylated DMRs, both the abundance and genomic coverage of RNA m6A modifications were significantly increased. This finding aligns with previous studies showing that stress can alter m6A methylation levels. For example, Yulan Zhao et al. observed that LPS activation induced global DNA hypomethylation accompanied by global m6A hypermethylation, with DNA 5mC and RNA m6A concurrently associated with up-regulation of PCK2 expression at the transcriptional and post-transcriptional levels, respectively [30]. Similarly, in a mouse model, exercise restored stress-induced decreases in brain m6A levels by elevating the methyl donor SAM, providing interventional evidence for the regulation of m6A by stress [31].

Genomic annotation of m6A peaks revealed a distribution pattern of exons > 3′ untranslated region (UTR) > 5′ UTR > first exon. This aligns with the canonical distribution of m6A methylation in eukaryotes, where this modification is predominantly enriched in exons and the 3′ UTR [32]. Enrichment of m6A peaks in exons may be involved in regulating RNA splicing [33], while their enrichment in the 3′ UTR can influence mRNA stability and translation efficiency [34]. In this study, the proportion of m6A peaks in both the 3′ UTR and 5′ UTR was slightly higher in the treated group compared to the control, suggesting that tail docking may regulate mRNA stability and translation by altering m6A modification levels in these regions. Functional enrichment analysis of genes associated with differential m6A peaks revealed their involvement in pathways such as signaling pathways regulating pluripotency of stem cells, regulation of the actin cytoskeleton, and endocytosis. These pathways are closely linked to the proliferation and differentiation potential of cells surrounding injury sites [35], tissue architecture remodeling [36], and substance transport. This indicates that tail docking-induced changes in m6A methylation may modulate energy balance, tissue repair, and overall metabolic stress response. Enrichment results revealed that the DEGs were significantly overrepresented in pathways related to stem cell pluripotency regulation and the Hippo signaling pathway. This pattern of enrichment is consistent with that observed for genes associated with both DMRs and differential m6A peaks. This convergence suggests that during the stress response to tail docking in sheep, DNA methylation and m6A methylation may occur in parallel or through associated mechanisms to influence downstream gene expression networks, thereby mediating the complex physiological response. Furthermore, analysis of gene expression levels (FPKM) showed a decrease in the number of low-expression genes (FPKM 0–0.5) and an increase in the number of moderately and highly expressed genes (FPKM ≥ 0.5) in the treated group. Given that FPKM > 0.5 is also used as a threshold for reliably expressed genes, this pattern suggests a potential link between tail docking and the activation of genomic transcriptional activity, possibly leading to the promoted expression of a broader set of genes. The Hippo signaling pathway was highly enriched in the functional analysis of both DEGs and DMR-associated genes. This pathway regulates cell proliferation, apoptosis [37], immune function, and differentiation [38], playing a crucial role in maintaining tissue homeostasis and facilitating repair processes following tail docking. In conclusion, tail docking stress appears to induce a comprehensive reprogramming of epigenetic and transcriptional regulation. The identification of a substantial set of DEGs provides robust, statistically grounded evidence for this genome-wide transcriptional response, offering a more direct and reliable measure of biological change than shifts in overall expression value distributions.

We identified 41 genes that exhibited concomitant alterations in both DNA and RNA methylation. These genes were primarily enriched in the adherens junction pathway. Tail docking is a potent stressor that triggers systemic physiological and molecular reprogramming [39]. The core repair processes—granulation tissue formation, re-epithelialization, and tissue remodeling [40]—heavily rely on cell migration and the dynamic re-establishment of intercellular connections [41]. This observation leads to a hypothesis that the systemic epigenetic reprogramming induced by tail-docking stress may target the adherens junction pathway as a key regulatory node. In this study, genes such as CITED4 and ZNF644 were found to be significantly altered following tail docking. CITED4 plays a critical role in stress responses [42] and represents a potential key node in the epigenetic regulatory network activated by tail docking. The traumatic insult of tail docking may modulate fat deposition and stress adaptation by activating relevant signaling pathways. Although a direct link between ZNF644 and tail docking in sheep has not been previously reported, our findings provide preliminary indication of its potential importance in this significant stress model. We hypothesize that CITED4 and ZNF644 may be involved in the response of fat-tailed sheep to tail-docking stress and could play important regulatory roles. Future studies should therefore focus on validating the specific functions of these two genes through functional assays, such as in vitro modulation (overexpression/knockdown) in adipocyte models or in vivo interventions, to systematically dissect their downstream targets and associated regulatory pathways, and thereby fully elucidate their mechanisms of action within the epigenetic reprogramming induced by tail docking. Finally, it is important to note some limitations of this study. Although efforts were made to control experimental conditions, potential confounding factors such as pen or batch effects cannot be entirely ruled out. Future studies employing larger cohorts and interventional approaches will be valuable to confirm these findings and elucidate the underlying mechanistic pathways.

This study systematically reveals the epigenetic reprogramming mechanisms underlying the response of fat-tailed sheep to tail docking. The response is characterized by a concurrent shift where hypomethylated DMRs predominated in the DNA methylome, coupled with RNA m6A hyper-methylation, which were linked to transcriptomic changes adapting to the stress. DMRs were significantly enriched in pathways related to tissue development and stress responses, such as the Hippo signaling pathway and adherens junction. This enrichment supports hypotheses that these pathways may be involved in tissue repair and stress adaptation processes following tail docking. Genes associated with differential m6A peaks were closely linked to processes including stem cell pluripotency and cytoskeleton regulation. We identified 41 genes with concurrent alterations in both DNA methylation and m6A methylation, predominantly enriched in the adherens junction pathway. Among these, CITED4 and ZNF644 were identified as prioritized candidate genes exhibiting consistent changes across three layers: DNA methylation, RNA m6A methylation, and gene expression. This work highlights the concomitant occurrence of DNA methylation and m6A methylation in mediating an organism’s response to environmental stimuli. It provides novel insights into the epigenetic regulation of phenotypic traits in livestock and establishes a theoretical foundation for optimizing the management of fat-tailed sheep through targeted epigenetic modulation.

## Figures and Tables

**Table 1:** Sequencing Data Preprocessing.

Sample	Raw Data		Valid Data		Valid%	Q20%	Q30%	GC%
	Read	Base	Read	Base				
C1	564794002	84.72G	542208536	72.72G	96.00	98.12	94.95	28.83
C2	561391610	84.21G	550890234	76.20G	98.13	98.08	94.83	27.34
C3	569096984	85.36G	560320256	75.89G	98.46	98.23	95.27	27.19
T1	572914584	85.94G	564069786	76.55G	98.46	98.15	95.11	27.07
T2	562752638	84.41G	556214190	76.66G	98.84	98.31	95.47	26.62
T3	584237704	87.64G	572415090	77.20G	97.98	98.28	95.38	28.92

**Table 2:** Statistical Results of Data Before and After Quality Preprocessing.

Sample	RawReads	RawBases	CleanReads	CleanBases	ValidBases	Q30	GC
C1	60.86M	9.13G	54.36M	8.15G	89.33%	93.08%	56.85%
C1_input	62.76M	9.41G	57.37M	8.61G	91.42%	88.78%	58.95%
C2	56.40M	8.46G	53.10M	7.96G	94.15%	92.97%	57.11%
C2_input	65.90M	9.88G	54.54M	8.18G	82.76%	89.20%	58.42%
C3	61.37M	9.21G	56.47M	8.47G	92.01%	92.64%	57.99%
C3_input	63.91M	9.59G	56.44M	8.47G	88.31%	86.48%	62.43%
T1	56.59M	8.49G	52.08M	7.81G	92.03%	93.15%	58.30%
T1_input	60.67M	9.10G	52.64M	7.90G	86.77%	88.97%	60.02%
T2	66.20M	9.93G	56.84M	8.53G	85.85%	93.26%	58.39%
T2_input	63.77M	9.57G	55.69M	8.35G	87.34%	88.30%	60.97%
T3	59.60M	8.94G	53.10M	7.96G	89.09%	92.48%	59.05%
T3_input	56.94M	8.54G	49.32M	7.40G	86.61%	84.99%	63.87%

**Table 3:** Peak Calling Statistics.

Sample Name	T_Group	C_Group
Percentage of Genome (%)	2.40	2.22
Median Length of Peaks (bp)	595	585
Average Length of Peaks (bp)	4092.80	4021.51
Number of Peaks	15,325	14,469
Length of Peaks (bp)	148—127,590	149—120,920

**Table 4:** Primer sequences for pyrosequencing.

Primer	Sequence (5′→3′)	Tm (°C)	Note
1F	GGTGTATGTTGGTGAGGGATTTA	60.5	Pyrosequencing
1R	AACCTTTAAACCCCAATCTATACCACCTA	59.5	203
2F	GGGGTTTGTTTATTTGTGTTGG	58.6	Pyrosequencing
2R	ACTACCTAAACCTTCCTTCTACCTAAATT	57.0	245
3F	GGTTTGTTTAGGGTGGGAGTATGTAT	60.2	Pyrosequencing
3R	ACAACACAATAACCCACTTAACAAATTCAA	58.3	189
4F	GATTGGATGATTGTTAATTGTGTAATGA	58.4	Pyrosequencing
4R	CCTACTCAAACCCCCACAAT	60.1	194
5F	GGTGGGGTTGTAGTTATTTATGAA	58.5	Pyrosequencing
5R	TTTCTCCCCCTCCATCCTCTAA	59.9	186
6F	GTTATTGGTTGTTTAATGATGAGTGAT	58.3	Pyrosequencing
6R	ACTCCCCTCCCATATCCCCAAATC	60.1	113
7F	GGTATGATGGTATAGGTGAGTAGT	59.7	Pyrosequencing
7R	ACCCCTAAAAAAACCAACCTAAA	59.1	273

**Table 5:** Statistics of Differentially Methylated Peaks.

Sample Name	T_Group-vs.-C_Group
Number of Peaks	2008
Total Length of Peaks (bp)	6,961,677
Average Length of Peaks (bp)	3466.97
Median Length of Peaks (bp)	448
Percentage of Genome (%)	0.27

**Table 6:** Primer sequence for RT-qPCR.

Primer	Sequence (5′→3′)	Tm (°C)	Note
METTL3-F	AACAATCTACTAAGGAACAA	60	RT-qPCR
METTL3-R	CAGAACTCTTGAACTTGA		136
METTL14-F	GGCTTCCTATGATACCTCTG	60	RT-qPCR
METTL14-R	TCCTTATATTCTTCCATCTTGTCT		90
WTAP-F	ATTCTTGTAATGCGACTA	60	RT-qPCR
WTAP-R	ATTGTTGATCTCAGTTGT		113
FTO-F	CTTGGAATACATCTTACAG	60	RT-qPCR
FTO-R	GATTTCAAAGAGATTTCAC		173
ALKBH5-F	TGGATACGCTGCTGATGA	60	RT-qPCR
ALKBH5-R	AGGATGATGACCGCTCTG		75
YTHDF1-F	GCATCAGAAGGATACCGTTCA	60	RT-qPCR
YTHDF1-R	TAACTGTTACTCTGGTTTGACTGT		75
YTHDF3-F	GGTCTCAGGATAAGTGGAA	60	RT-qPCR
YTHDF3-R	CTAAGCGAATATGCCGTAAT		90
Actin-F	TTCTAGGCGGACTGTTAG	60	RT-qPCR
Actin-R	TGCCAATCTCATCTCGTT		80

## Data Availability

The raw data that support the findings of this study are accessible from the corresponding author, subject to a formal and reasonable request.

## Supplementary Materials

The following supporting information can be downloaded at: https://www.mdpi.com/article/10.3390/ani16030481/s1, Figure S1. Per-base sequence quality profile of the raw WGBS sequencing data assessed by FastQC. The x-axis indicates the position in the read (bp), and the y-axis shows the base calling error rate. Figure S2. Coverage distribution of CpG sites across different sequencing depths for the WGBS samples. Figure S3. M-bias plot showing the methylation rate as a function of read position for WGBS libraries. Table S1. Summary of alignment and conversion metrics for WGBS data. Table S2. Categorization and counts of predicted RNA m6A methylation sites by confidence level. Table S3. Pyrosequencing-derived methylation levels (%) for individual CpG sites within the seven assays of the DGAT1 promoter region. Table S4. Distribution of gene counts across FPKM expression intervals. Figures S4–S10. Pyrosequencing validation of CpG site methylation across seven assays (Assay 1S–7S) in the promoter region of DGAT1. Figure S11. KEGG enrichment analysis of the top 30 genes associated with differential m6A methylation peaks in the control group. Figure S12. KEGG enrichment analysis of the top 30 genes associated with differential m6A methylation peaks in the tail-docked (experimental) group. Figure S13. KEGG enrichment analysis of genes associated with differential RNA m6A methylation peaks (T_Group vs. C_Group). Figure S14. KEGG enrichment analysis of differentially expressed genes (DEGs) between the tail-docked and control groups.
