# Supplementary material for: Concurrent Alterations in DNA Methylation and RNA m6A Methylation During Epigenetic and Transcriptomic Reprogramming Induced by Tail Docking Stress in Fat-Tailed Sheep
Source: Animals (Basel). 2026 Feb 4;16(3):481. doi: 10.3390/ani16030481 (PMC12896734; doi:10.3390/ani16030481)

CpG Pyrogram Report Run: 20250319 A

Well: D7
Assay: 1S
Sample ID: 1
Sequence Before Bisulfite Treatment: -
Sequence to analyze:
YGGTTTGGGTGGGTTAGGGATGGTTATAYGTGTAGAGAGAGTYGYGGTTTTTAGATTTAAGYGTGGGTTTTTTATTTTTTTTTATTT
Analysis parameters have been edited.

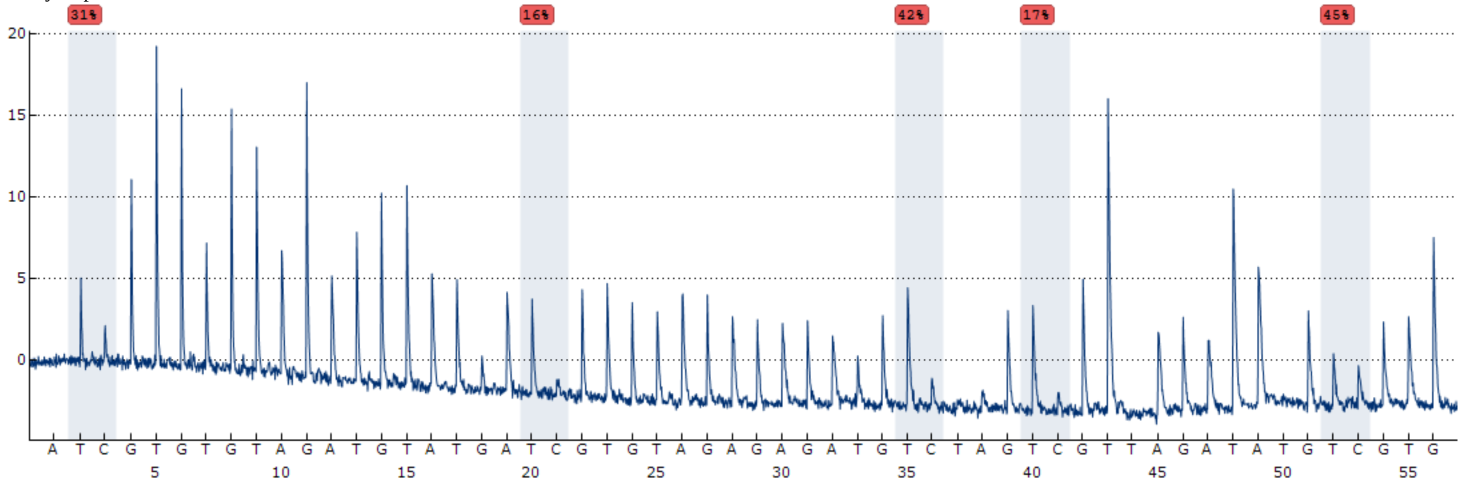

Well: D8
Assay: 1S
Sample ID: 2
Sequence Before Bisulfite Treatment: -
Sequence to analyze:
YGGTTTGGGTGGGTTAGGGATGGTTATAYGTGTAGAGAGAGTYGYGGTTTTTAGATTTAAGYGTGGGTTTTTTATTTTTTTTTATTT
Analysis parameters have been edited.

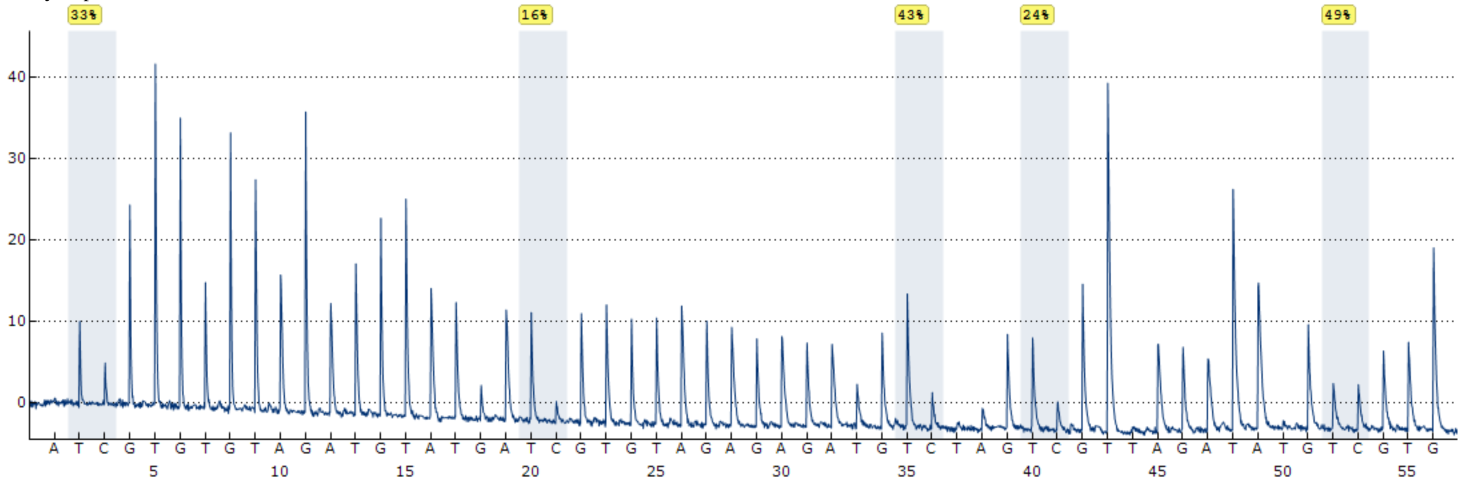

Well: D9
Assay: 1S
Sample ID: 3
Sequence Before Bisulfite Treatment: -
Sequence to analyze:
YGGTTTGGGTGGGTTAGGGATGGTTATAYGTGTAGAGAGAGTYGYGGTTTTTAGATTTAAGYGTGGGTTTTTTATTTTTTTTTATTT
Analysis parameters have been edited.

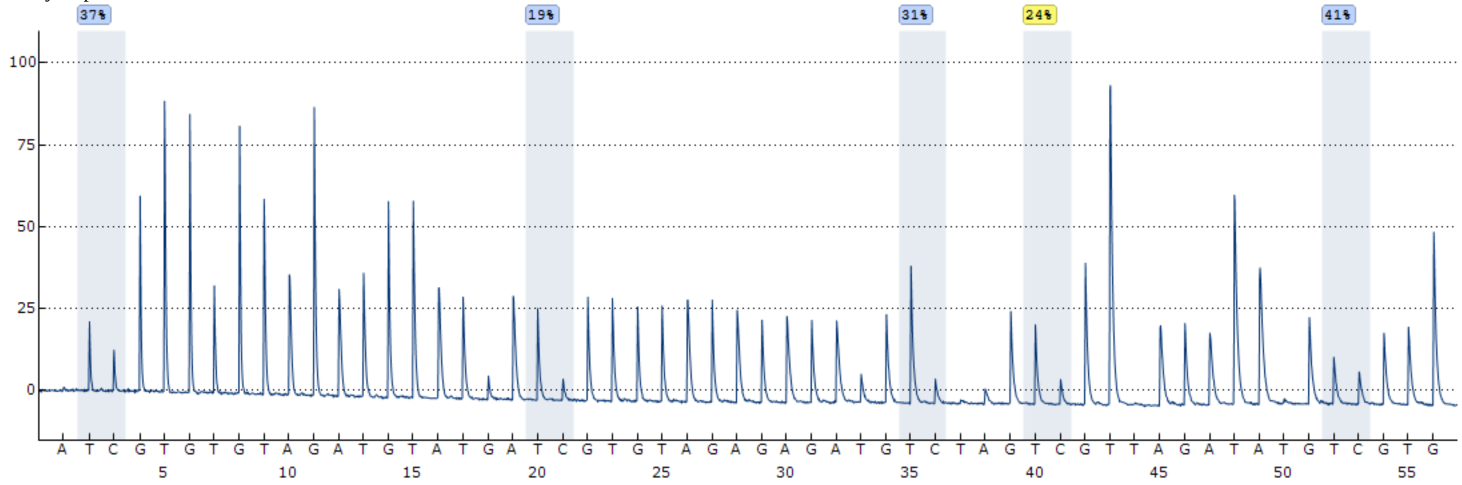

CpG Pyrogram Report Run: 20250319 A

Well: D10
Assay: 1S
Sample ID: 4
Sequence Before Bisulfite Treatment: -
Sequence to analyze:
YGGTTTGGGTGGGTTAGGGATGGTTATAYGTGTAGAGAGAGTYGYGGTTTTTAGATTTAAGYGTGGGTTTTTTATTTTTTTTTATTT
Analysis parameters have been edited.

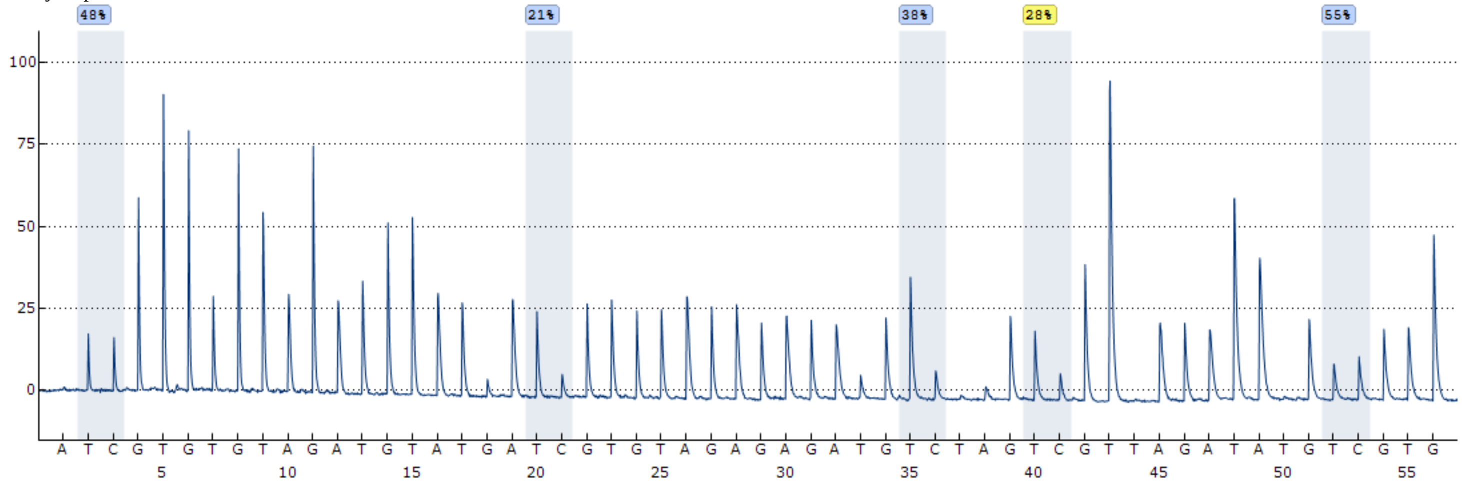

Well: D11
Assay: 1S
Sample ID: 5
Sequence Before Bisulfite Treatment: -
Sequence to analyze:
YGGTTTGGGTGGGTTAGGGATGGTTATAYGTGTAGAGAGAGTYGYGGTTTTTAGATTTAAGYGTGGGTTTTTTATTTTTTTTTATTT
Analysis parameters have been edited.

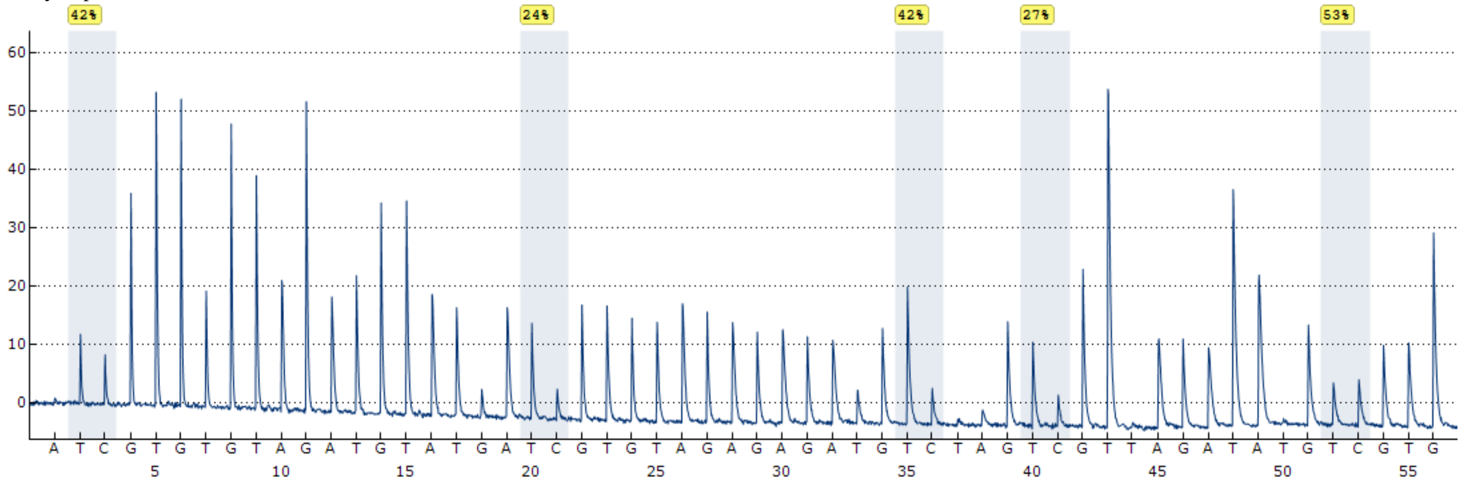

Well: D12
Assay: 1S
Sample ID: 6
Sequence Before Bisulfite Treatment: -
Sequence to analyze:
YGGTTTGGGTGGGTTAGGGATGGTTATAYGTGTAGAGAGAGTYGYGGTTTTTAGATTTAAGYGTGGGTTTTTTATTTTTTTTTATTT
Analysis parameters have been edited.

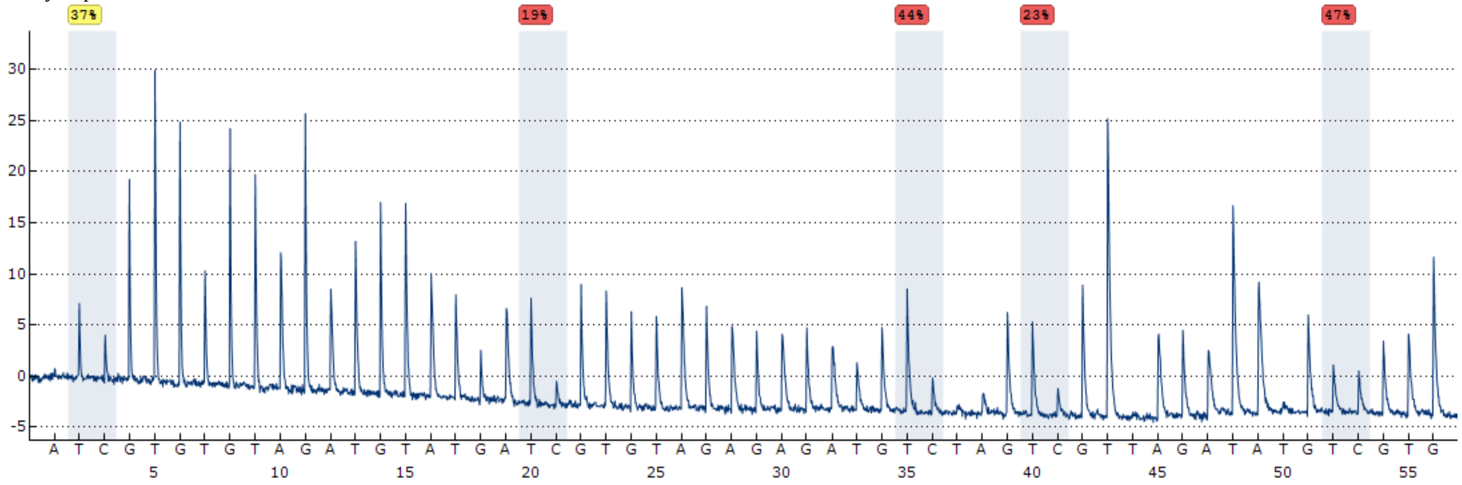

Supplement: Supplementary file 1 [file animals-16-00481-s001.zip › Supplementary Materials/Supplemental Figure S4.pdf]
