# Supplementary material for: Concurrent Alterations in DNA Methylation and RNA m6A Methylation During Epigenetic and Transcriptomic Reprogramming Induced by Tail Docking Stress in Fat-Tailed Sheep
Source: Animals (Basel). 2026 Feb 4;16(3):481. doi: 10.3390/ani16030481 (PMC12896734; doi:10.3390/ani16030481)

CpG Pyrogram Report Run: 20250317 B

Well: B1
Assay: 4S
Sample ID: 1
Sequence Before Bisulfite Treatment: -
Sequence to analyze:
GYGGGGAGTTTTGTAAGTTTGYGGATGAAGGTAGAAGYGGGGYGGGTGGAGAGGYGGGTTGGYGTGTTTATTGTGGGGGTTTGAGTAG
Analysis parameters have been edited.

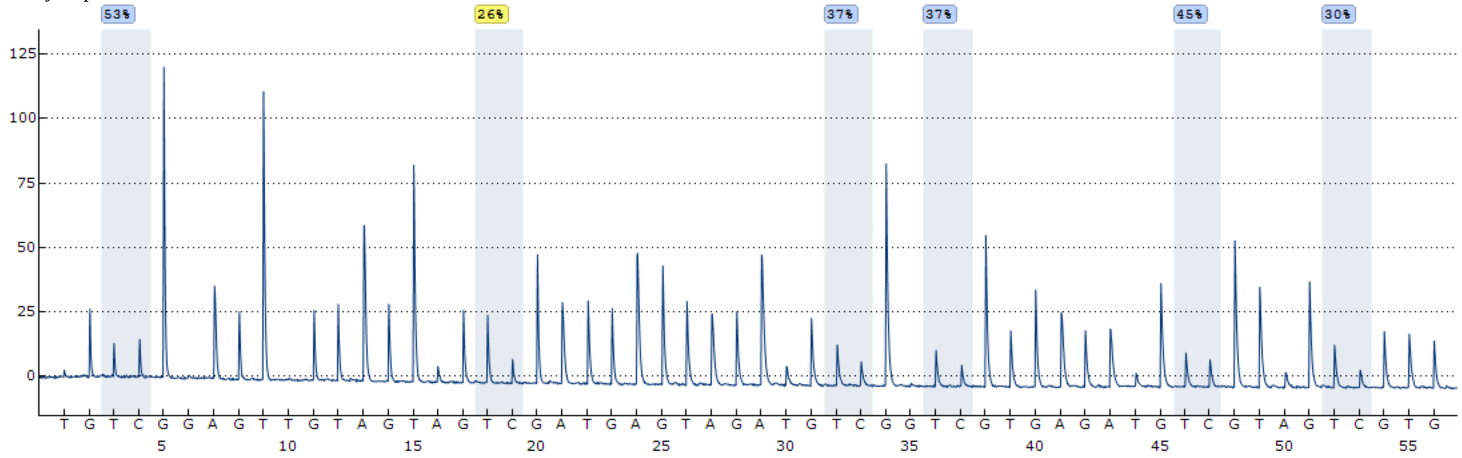

Well: B2
Assay: 4S
Sample ID: 2
Sequence Before Bisulfite Treatment: -
Sequence to analyze:
GYGGGGAGTTTTGTAAGTTTGYGGATGAAGGTAGAAGYGGGGYGGGTGGAGAGGYGGGTTGGYGTGTTTATTGTGGGGGTTTGAGTAG
Analysis parameters have been edited.

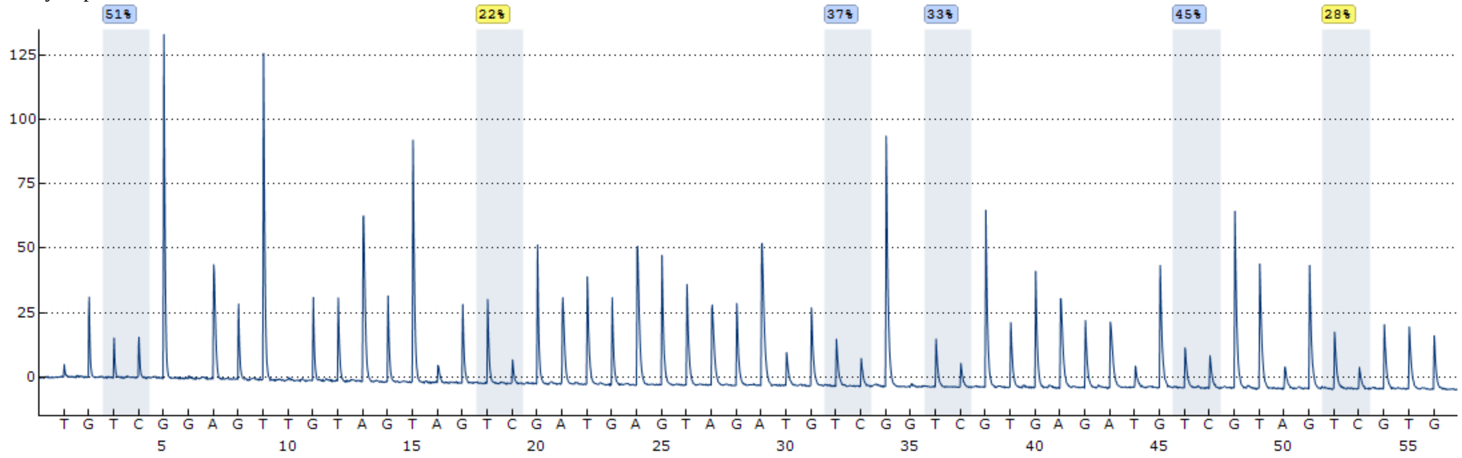

Well: B3
Assay: 4S
Sample ID: 3
Sequence Before Bisulfite Treatment: -
Sequence to analyze:
GYGGGGAGTTTTGTAAGTTTGYGGATGAAGGTAGAAGYGGGGYGGGTGGAGAGGYGGGTTGGYGTGTTTATTGTGGGGGTTTGAGTAG
Analysis parameters have been edited.

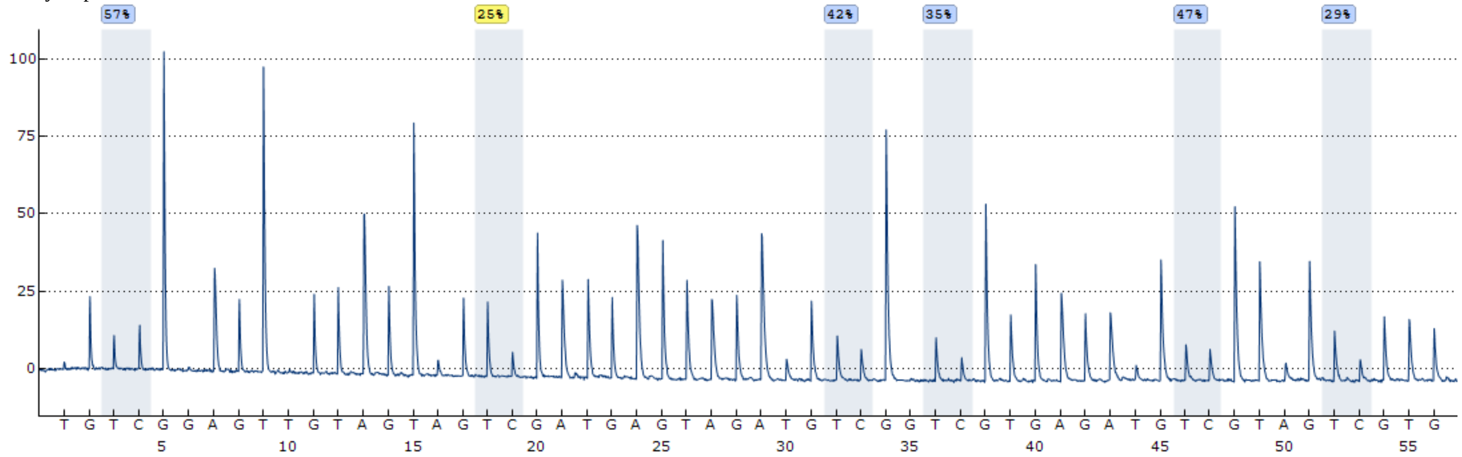

CpG Pyrogram Report Run: 20250317 B

Well: B4
Assay: 4S
Sample ID: 4
Sequence Before Bisulfite Treatment: -
Sequence to analyze:
GYGGGGAGTTTTGTAAGTTTGYGGATGAAGGTAGAAGYGGGGYGGGTGGAGAGGYGGGTTGGYGTGTTTATTGTGGGGGTTTGAGTAG
Analysis parameters have been edited.

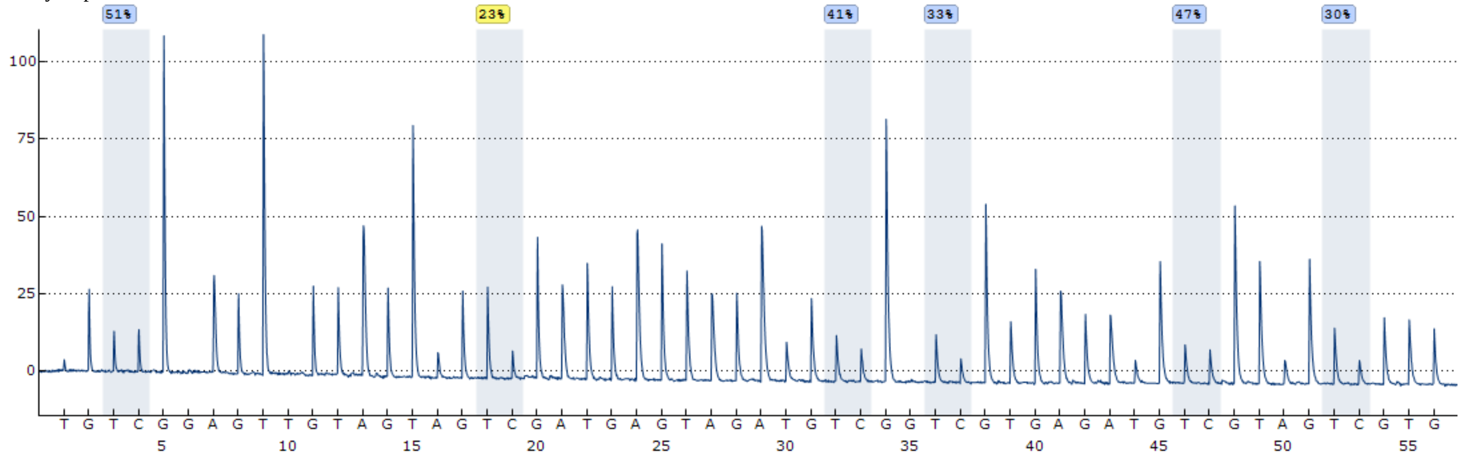

Well: B5
Assay: 4S
Sample ID: 5
Sequence Before Bisulfite Treatment: -
Sequence to analyze:
GYGGGGAGTTTTGTAAGTTTGYGGATGAAGGTAGAAGYGGGGYGGGTGGAGAGGYGGGTTGGYGTGTTTATTGTGGGGGTTTGAGTAG
Analysis parameters have been edited.

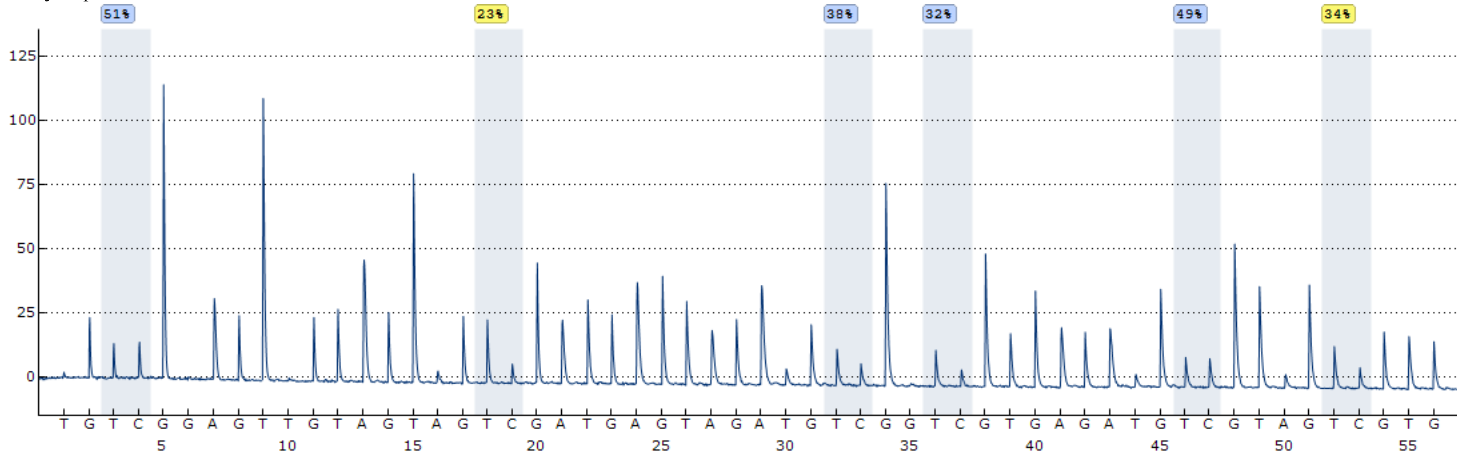

Well: B6
Assay: 4S
Sample ID: 6
Sequence Before Bisulfite Treatment: -
Sequence to analyze:
GYGGGGAGTTTTGTAAGTTTGYGGATGAAGGTAGAAGYGGGGYGGGTGGAGAGGYGGGTTGGYGTGTTTATTGTGGGGGTTTGAGTAG
Analysis parameters have been edited.

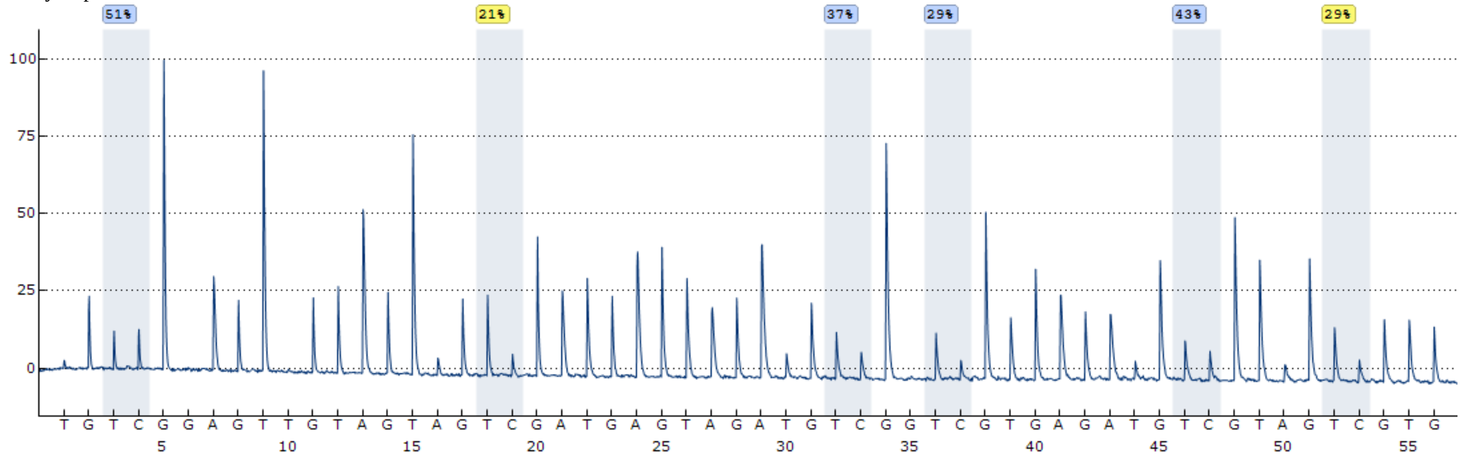

Supplement: Supplementary file 1 [file animals-16-00481-s001.zip › Supplementary Materials/Supplemental Figure S7.pdf]
