# Supplementary material for: Concurrent Alterations in DNA Methylation and RNA m6A Methylation During Epigenetic and Transcriptomic Reprogramming Induced by Tail Docking Stress in Fat-Tailed Sheep
Source: Animals (Basel). 2026 Feb 4;16(3):481. doi: 10.3390/ani16030481 (PMC12896734; doi:10.3390/ani16030481)
Supplement: Supplementary file 1 [file animals-16-00481-s001.zip › Supplementary Materials/Supplemental Table S1.pdf]

Sample	Total reads	Mapped reads	Mapping rate(%)	Duplication rate(%)	BS conversion rate(%)
C1	542208536	391883418	72.28	9.19	99.43
C2	550890234	413715088	75.10	8.51	99.42
C3	560320256	416532796	74.34	9.57	99.46
T1	564069786	424619808	75.28	8.37	99.46
T2	556214190	425557008	76.51	9.59	99.46
T3	572415090	415682342	72.62	9.98	99.45
