# Supplementary material for: Concurrent Alterations in DNA Methylation and RNA m6A Methylation During Epigenetic and Transcriptomic Reprogramming Induced by Tail Docking Stress in Fat-Tailed Sheep
Source: Animals (Basel). 2026 Feb 4;16(3):481. doi: 10.3390/ani16030481 (PMC12896734; doi:10.3390/ani16030481)
Supplement: Supplementary file 1 [file animals-16-00481-s001.zip › Supplementary Materials/Supplemental Table S3.pdf]

Item / CpG(%)	Assay 1S	Assay 2S	Assay 3S	Assay 4S	Assay 5S	Assay 6S	Assay 7S
C_group (Mean ± SD)	31.20±11.38	84.89±7.71	81.26±19.92	37.72±10.29	33.00±9.42	62.83±12.96	65.62±13.73
T_group (Mean ± SD)	36.53±12.02	84.83±6.49	79.41±12.12	36.78±10.17	31.67±10.24	62.11±13.50	63.57±17.18
