# Supplementary material for: Concurrent Alterations in DNA Methylation and RNA m6A Methylation During Epigenetic and Transcriptomic Reprogramming Induced by Tail Docking Stress in Fat-Tailed Sheep
Source: Animals (Basel). 2026 Feb 4;16(3):481. doi: 10.3390/ani16030481 (PMC12896734; doi:10.3390/ani16030481)
Supplement: Supplementary file 1 [file animals-16-00481-s001.zip › Supplementary Materials/Supplemental table S2.pdf]

Classification	C_Group	T_Group
Very high	30706	32484
Non	857579	926863
Moderate	89741	95254
Low	76617	81508
High	96480	101739
