# Supplementary material for: Concurrent Alterations in DNA Methylation and RNA m6A Methylation During Epigenetic and Transcriptomic Reprogramming Induced by Tail Docking Stress in Fat-Tailed Sheep
Source: Animals (Basel). 2026 Feb 4;16(3):481. doi: 10.3390/ani16030481 (PMC12896734; doi:10.3390/ani16030481)
Supplement: Supplementary file 1 [file animals-16-00481-s001.zip › Supplementary Materials/Supplemental table S4.pdf]

sample	FPKM >=10	FPKM 1–10	FPKM 0.5–1	FPKM 0–0.5
T1_input	5334	6814	1164	7207
T2_input	4956	6799	1253	7511
T3_input	4929	6582	1296	7712
C1_input	4651	6626	1190	8052
C2_input	5469	6483	1059	7508
C3_input	4787	6490	1219	8023
