# Supplementary figures and images for: Concurrent Alterations in DNA Methylation and RNA m6A Methylation During Epigenetic and Transcriptomic Reprogramming Induced by Tail Docking Stress in Fat-Tailed Sheep
Source: Animals (Basel). 2026 Feb 4;16(3):481. doi: 10.3390/ani16030481 (PMC12896734; doi:10.3390/ani16030481)

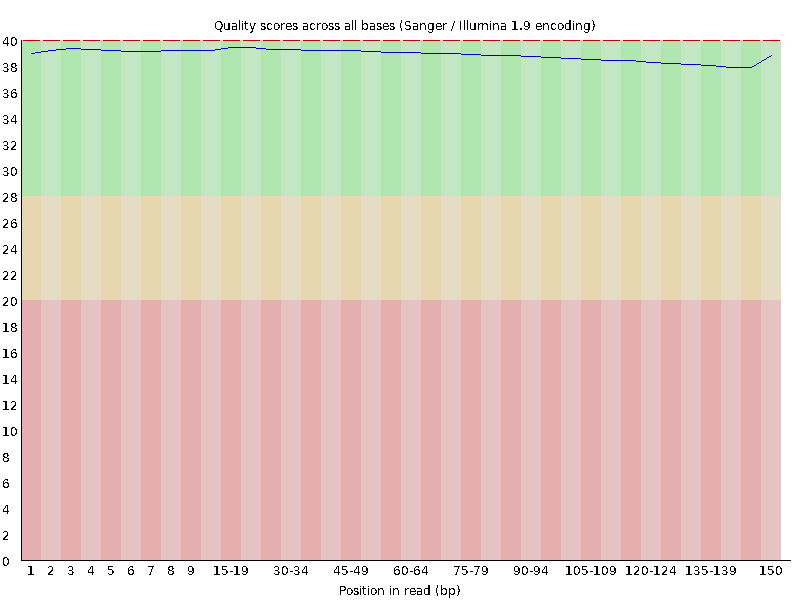

Supplement: Supplementary file 1 [file animals-16-00481-s001.zip › Supplementary Materials/Supplemental Figure S1.png]

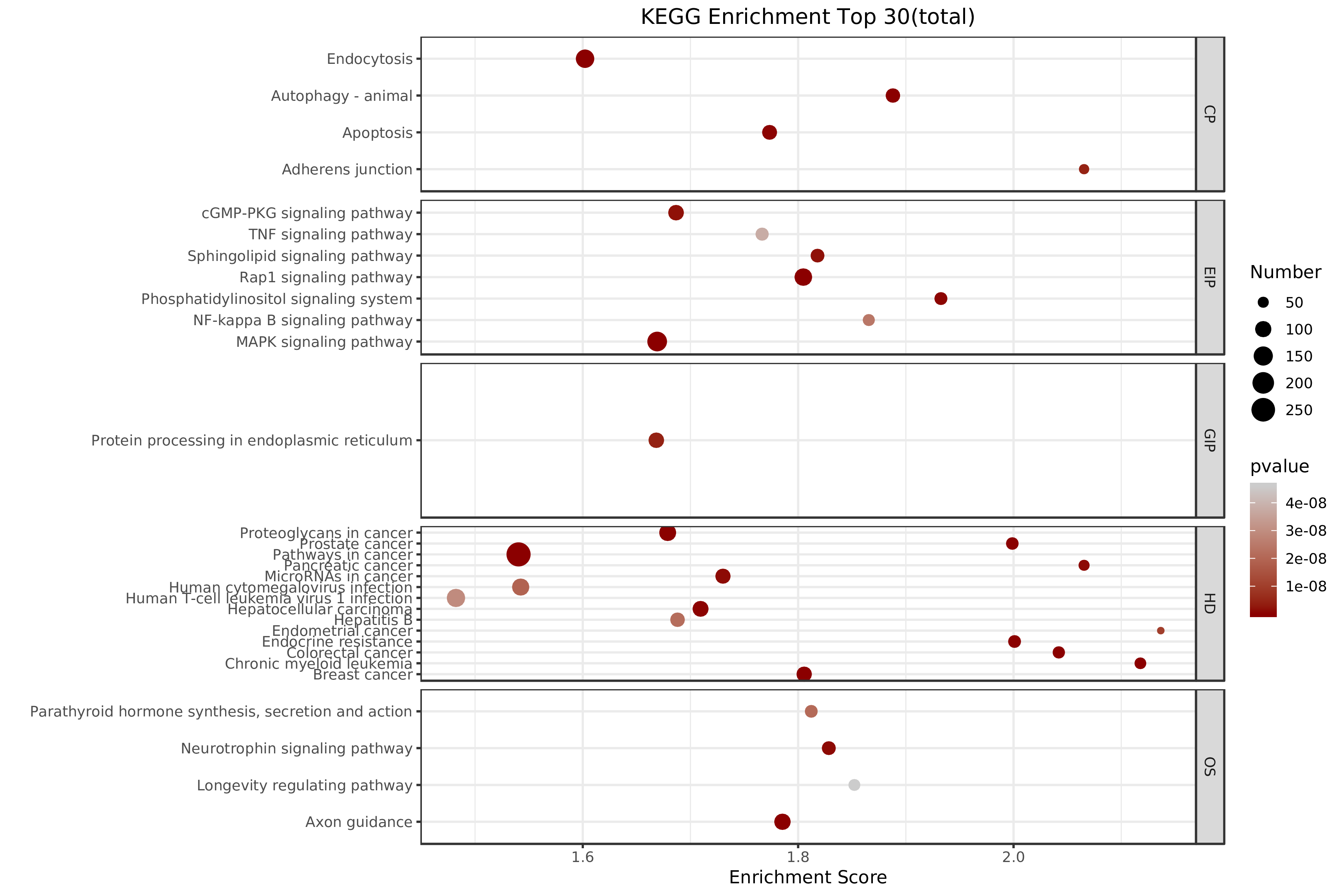

Supplement: Supplementary file 1 [file animals-16-00481-s001.zip › Supplementary Materials/Supplemental Figure S11.png]

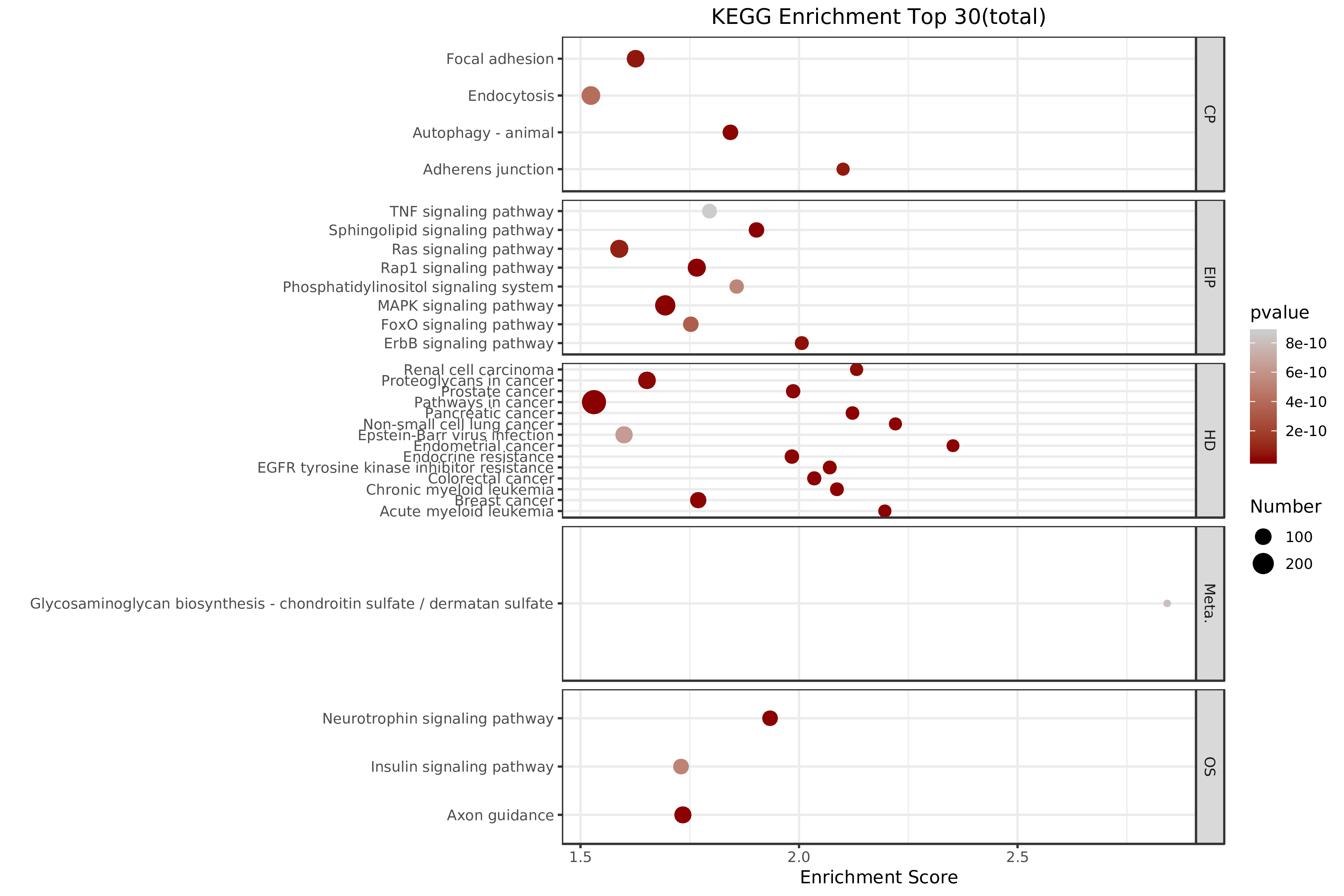

Supplement: Supplementary file 1 [file animals-16-00481-s001.zip › Supplementary Materials/Supplemental Figure S12.png]

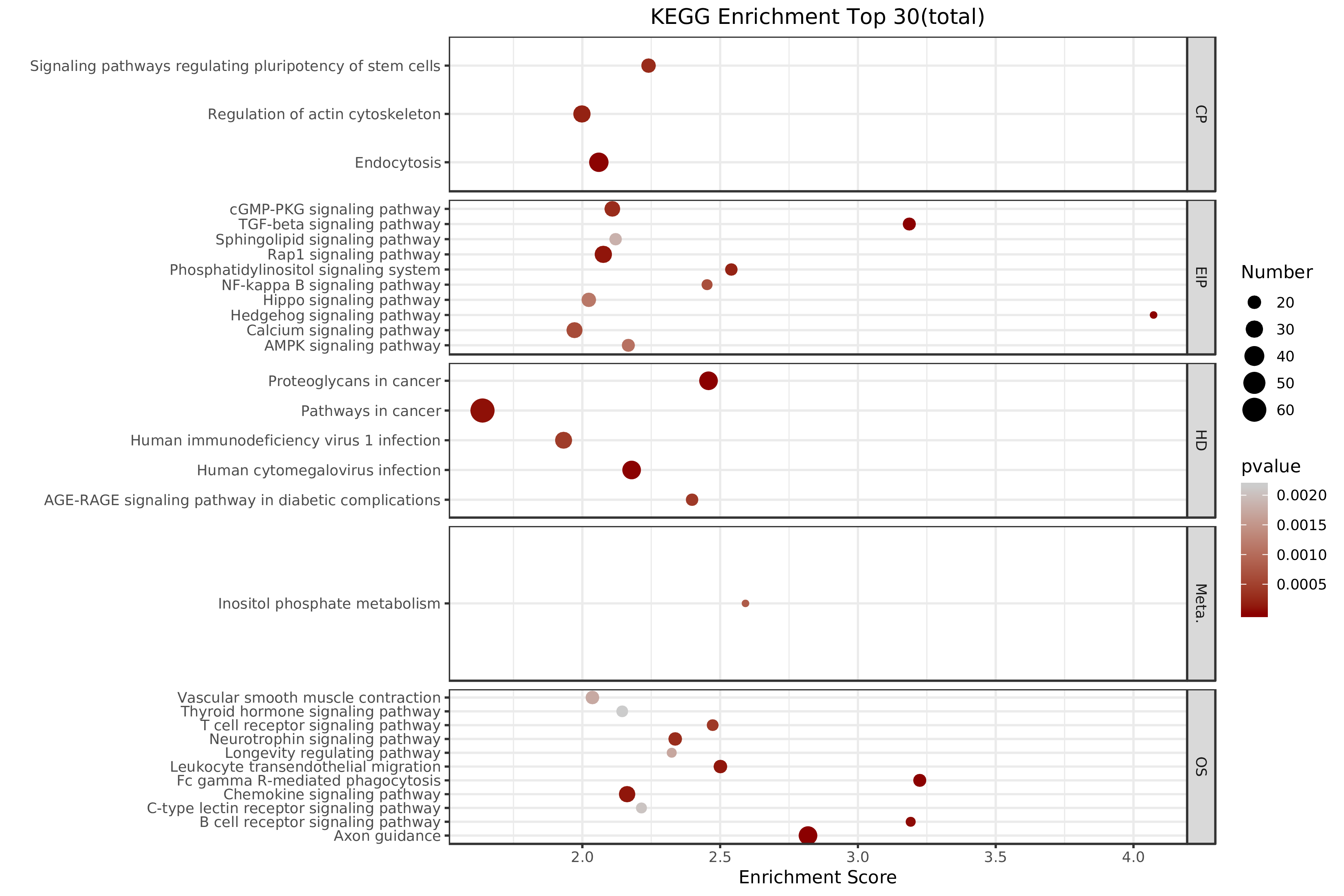

Supplement: Supplementary file 1 [file animals-16-00481-s001.zip › Supplementary Materials/Supplemental Figure S13.png]

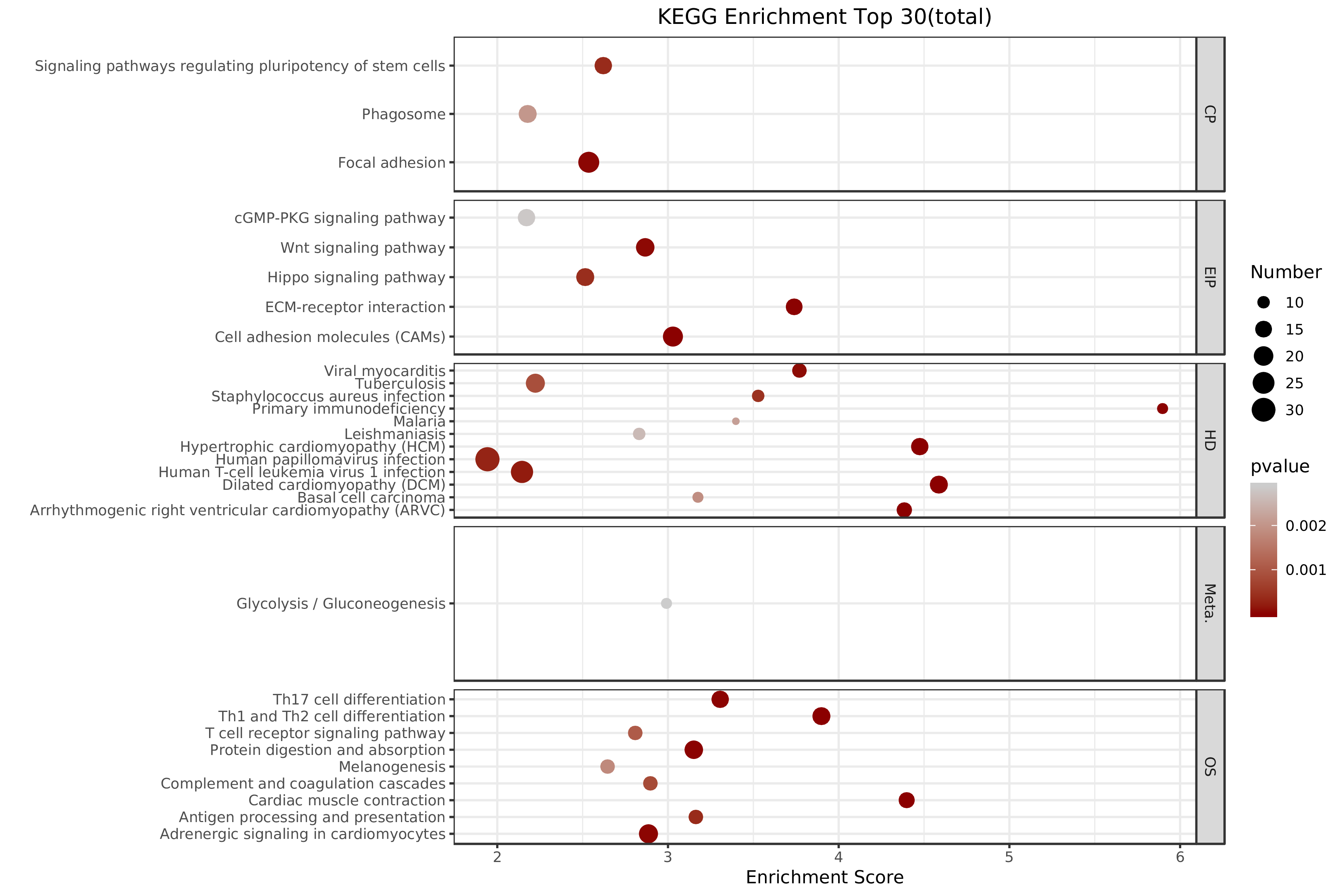

Supplement: Supplementary file 1 [file animals-16-00481-s001.zip › Supplementary Materials/Supplemental Figure S14.png]

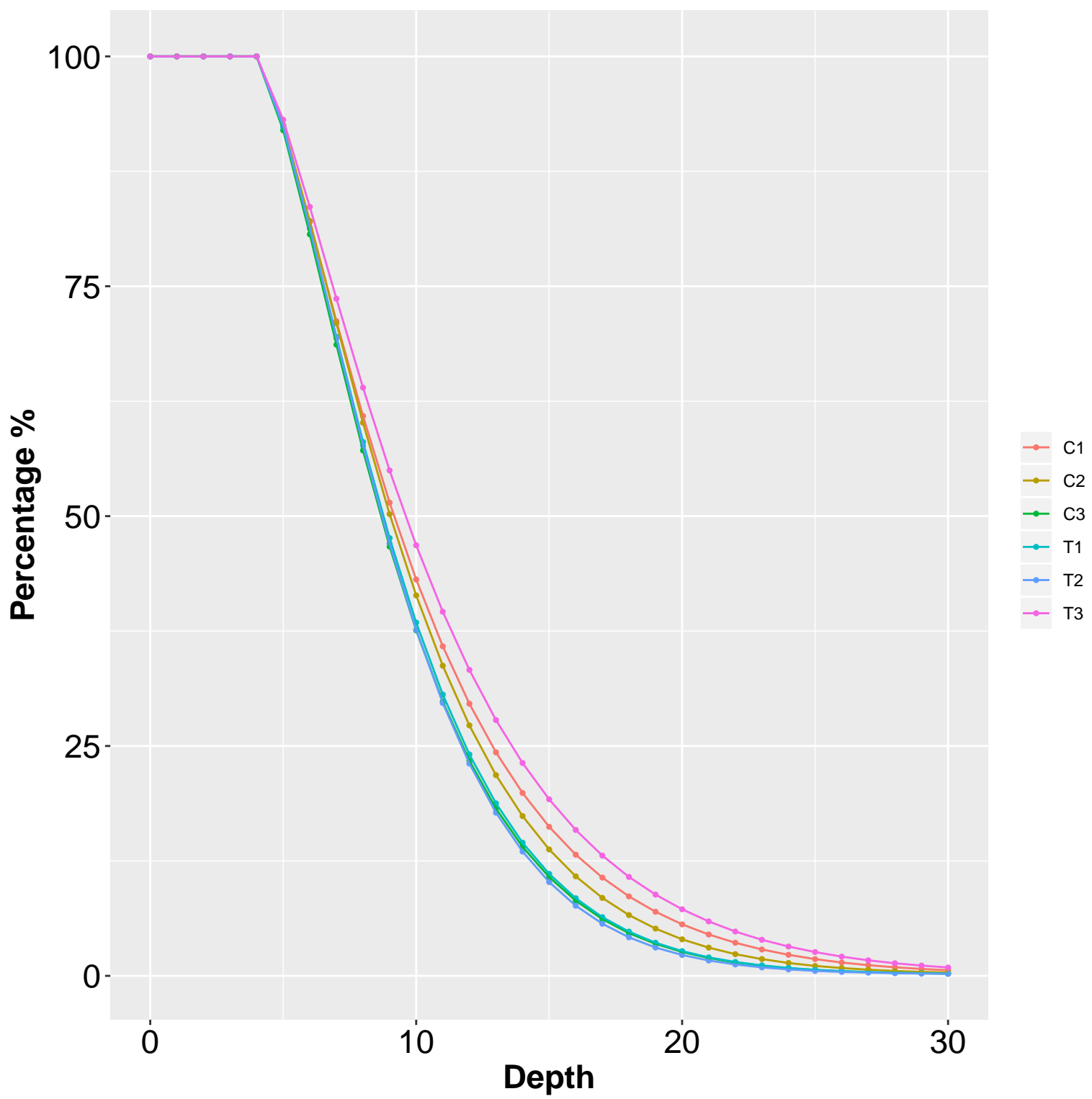

Supplement: Supplementary file 1 [file animals-16-00481-s001.zip › Supplementary Materials/Supplemental Figure S2.pdf]

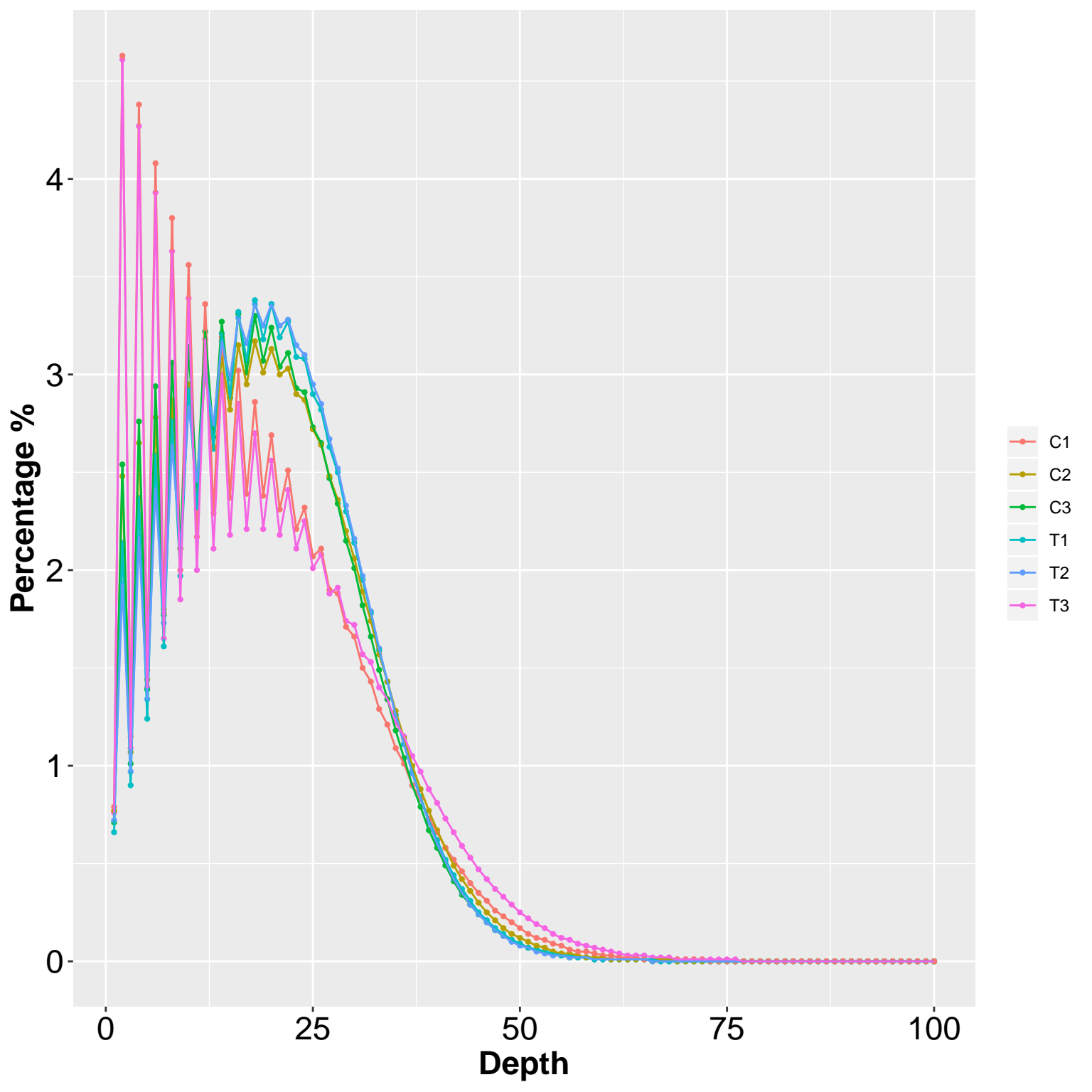

Supplement: Supplementary file 1 [file animals-16-00481-s001.zip › Supplementary Materials/Supplemental Figure S3.pdf]
